# Unveiling Disrupted Lipid Metabolism in Benign Prostate Hyperplasia, Prostate Cancer, and Metastatic Patients: Insights from a Colombian Nested Case–Control Study

**DOI:** 10.3390/cancers15225465

**Published:** 2023-11-18

**Authors:** Daniel Pardo-Rodriguez, Mary Santamaría-Torres, Angela Salinas, Eliécer Jiménez-Charris, Mildrey Mosquera, Mónica P. Cala, Herney Andrés García-Perdomo

**Affiliations:** 1 Metabolomics Core Facility—MetCore, Vice-Presidency for Research, Universidad de los Andes, Bogotá 110111, Colombia; d.pardorodriguez@uniandes.edu.co (D.P.-R.); m.santamariatorres@uniandes.edu.co (M.S.-T.); 2 Grupo de Nutrición, Departamento de Ciencias Fisiológicas, Facultad de Salud, Universidad del Valle, Cali 760043, Colombia; angela.salinas@correounivalle.edu.co (A.S.); eliecer.jimenez@correounivalle.edu.co (E.J.-C.); mildrey.mosquera@correounivalle.edu.co (M.M.); 3 UROGIV Research Group, School of Medicine, Universidad del Valle, Cali 72824, Colombia; 4 Division of Urology/Urooncology, Department of Surgery, School of Medicine, Universidad del Valle, Cali 72824, Colombia

**Keywords:** benign prostatic hyperplasia, Colombia, lipidic alteration, prostate cancer, metastasis, untargeted metabolomics analysis

## Abstract

**Simple Summary:**

Prostate cancer represents a substantial global health issue, and its intricacies continue to pose challenges for complete comprehension. In this study, an untargeted metabolomic and lipidomic approach was employed to analyze plasma samples obtained from patients diagnosed with benign prostatic hyperplasia (BPH), prostate cancer (PCa), and metastatic prostate cancer (Met). The results revealed significant changes in lipid metabolism when comparing patients with prostate cancer to those with benign prostatic hyperplasia and patients with metastasis. These findings support the fundamental importance of lipid metabolism in the development and progression of prostate cancer.

**Abstract:**

Prostate cancer is a significant global health concern, and its prevalence is increasing worldwide. Despite extensive research efforts, the complexity of the disease remains challenging with respect to fully understanding it. Metabolomics has emerged as a powerful approach to understanding prostate cancer by assessing comprehensive metabolite profiles in biological samples. In this study, metabolic profiles of patients with benign prostatic hyperplasia (BPH), prostate cancer (PCa), and metastatic prostate cancer (Met) were characterized using an untargeted approach that included metabolomics and lipidomics via liquid chromatography and gas chromatography coupled with high-resolution mass spectrometry. Comparative analysis among these groups revealed distinct metabolic profiles, primarily associated with lipid biosynthetic pathways, such as biosynthesis of unsaturated fatty acids, fatty acid degradation and elongation, and sphingolipid and linoleic acid metabolism. PCa patients showed lower levels of amino acids, glycerolipids, glycerophospholipids, sphingolipids, and carnitines compared to BPH patients. Compared to Met patients, PCa patients had reduced metabolites in the glycerolipid, glycerophospholipid, and sphingolipid groups, along with increased amino acids and carbohydrates. These altered metabolic profiles provide insights into the underlying pathways of prostate cancer’s progression, potentially aiding the development of new diagnostic, and therapeutic strategies.

## 1. Introduction

Prostate cancer (PCa) is a prevalent cancer affecting males and contributes considerably to increased mortality rates globally. PCa patients might present with either localized or advanced illness. PCa showed the highest prevalence among males over 45 years old in 2020, with 1,409,428 new cases, and placed sixth in terms of death (374,018), ahead of liver, colorectal, and stomach cancers. During the same year in Colombia, prostate cancer had the greatest incidence with 14,422 cases and the highest mortality rate with 3835 deaths [1].

PCa is characterized by a moderate rate of development and early asymptomaticism. This in turn makes early diagnosis difficult [2]. PCa can be diagnosed using a variety of techniques, including transrectal ultrasound, digital rectal examination, and biopsy. These techniques, however, are intrusive and could make patients feel uncomfortable or ashamed [3]. Prostate-specific antigen (PSA) testing is another diagnostic technique. The limited specificity of this biomarker is highlighted by the fact that a sizable fraction of individuals with benign diseases, such as inflammation or hyperplasia, may have abnormal PSA levels, leading to unnecessary biopsies [4]. As a result, several expert groups, including the American Cancer Society, suggest that the current data on the effectiveness of PSA screening are insufficient to support its routine use [5]. This demands research on novel approaches to prostate cancer detection.

On the other hand, intrinsic factors, such as genetic alterations and origin tissue, as well as extrinsic factors, such as access to nutrients and oxygen, interactions with cells in the microenvironment, and radiation or chemotherapy exposure, may all impact the onset and development of the tumor environment [6]. This suggests that population-specific factors, such as race, genetics, dietary patterns, and lifestyle, influence the possibility of developing the disease or the disease progressing, as well as the recurrence and mortality rates for certain types of cancer [6,7,8,9]. For example, prostate cancer is uncommon among Asian males, with age-adjusted incidence rates ranging from 2 to 10 per 100,000 people. Northern Europe, on the other hand, has a high incidence rate, whereas African-American men have the highest incidence rate worldwide [10]. All of this requires an in-depth knowledge of cancer and its progression in patient cohorts from different geographical areas.

Metabolic changes in biofluids reflect changes in an individual’s physiological state, making it a useful tool for understanding disease-related physiological processes and identifying noninvasive biomarkers. Recent research has shown that changes in metabolism have an impact on cancer development [11,12]. The integration of untargeted metabolomics and advanced informatics has the potential to reveal the intricate metabolic complexity of living systems [13,14,15]. Thus, the current proposal aimed to establish differential profiles among patients with benign prostatic hyperplasia (BPH), PCa, and metastatic prostate cancer (Met) through a case–control research nested in Colombia. To our knowledge, this is one of the first studies to be carried out in a cohort of Colombian patients. This type of research will establish the groundwork for understanding PCa in the Colombian population and may identify metabolites that could be used as possible markers for the disease’s diagnosis in the future.

## 2. Materials and Methods

### 2.1. Study Participants

We conducted a case–control study between November 2019 to November 2020. The patients were recruited from the outpatient clinic and the prostate biopsy section of the Urology Department at the Universidad del Valle and the Hospital Universitario del Valle, Evaristo Garcia. This study was accepted by the ethics and research committee of the hospital, under code 074-2019. The study included 37 individuals with BPH, 34 with PCa, 17 with confirmed Met, and 20 young individuals (HV) as a control group. The first cohort consisted of participants aged 40 years or older with a diagnosis of localized PCa. The risk classification of these patients was determined based on the categories defined by the European Association of Urology [16]. In addition to the older age group, a second cohort consisted of participants with benign prostatic hyperplasia (BPH). The study also enrolled patients with confirmed metastasis (Met) through imaging methods or those undergoing chemotherapy or radiotherapy as a palliative approach for advanced disease and healthy volunteers (HV), aged between 25 and 35 years, without a personal history of genitourinary tract disease. These individuals did not exhibit the conditions described in the exclusion criteria.

On the other hand, exclusion criteria included the following: patients with the concomitant presence of other types of cancer, coagulation disorders, renal disorders, or metabolic disorders, such as diabetes mellitus, gout, or hyperthyroidism; individuals with symptoms of acute illnesses within two weeks prior to sample collection, such as fever, cough, headache, diarrhea, hematuria, as well as psychiatric disorders or episodes of stress-related trauma; and the use of certain medications within two weeks before sample collection, including antibiotics, hormones, nonsteroidal anti-inflammatory drugs, and chemotherapy or radiotherapy medications, as well as failure to provide signed informed consent. After obtaining signed consent, the blood samples from each patient were centrifuged for 5 min at 4000 rpm at 4 °C. Following centrifugation, the plasma layer was carefully collected and then stored at −80 °C to maintain its integrity. Subsequently, the samples were transported to the Metabolomics Core Facility at the University of Los Andes for further analysis.

### 2.2. Metabolomics Analysis by RP-LC-QTOF-MS

A total of 100 µL of plasma was utilized for the extraction procedure, in which 300 µL of cold methanol (−20 °C) was added and vortexed at 3200 rpm for 3 min. The samples were then allowed to stand at −20 °C for 20 min to precipitate proteins and were subsequently subjected to centrifugation at 13,000 rpm, 4 °C for 10 min. The supernatant was collected and preserved for analysis using RP-LC-QTOF-MS and GC-QTOF-MS. The samples were analyzed using an Agilent Infinity 1260 liquid chromatography system coupled to an Agilent 6545 quadrupole time-of-flight mass spectrometer analyzer with electrospray ionization (Agilent Jet Stream ESI source). A total of 2 µL of the sample was injected into a ZORBAX Eclipse Plus C18 column (50 mm × 2.1 mm, 1.8 µm; Agilent, Santa Clara, CA, USA) at 60 °C. The mobile phase employed was composed of 0.1% (*v*/*v*) formic acid in Type I ultrapure water (Phase A) and 0.1% (*v*/*v*) formic acid in acetonitrile (Phase B) with a constant flow rate of 0.6 mL/min. The elution gradient was programmed with the following specifications: 5% B for 1 min; 5–80% B in 6 min; 100% B for 4.5 min. Finally, the flow rate was decreased to 5% of Phase B and held for 5 min until the equipment was reconditioned. Mass spectrometry detection was performed in positive electrospray ionization mode in full-scan mode from 100 to 1100 *m*/*z*. The QTOF instrument was operated in 4 GHz (high resolution) mode. Two reference masses, *m*/*z* 121.0509 [purine, ([C_5_H_4_N_4_+H]^+^) and *m*/*z* 922.0098 [HP-0921, ([C_18_H_18_O_6_N_3_P_3_F_24_+H]^+^), were used for mass correction in positive mode throughout the analysis. MS/MS acquisition mode was performed by data-dependent acquisition mode using a QC sample at different collision energies of 20 and 40 eV.

### 2.3. Metabolomics Analysis by GC-QTOF-MS

From the previously prepared metabolic extracts, 50 µL was dried in a SpeedVac (Thermo Scientific, Waltham, MA, USA) for 1 h at 35 °C. Then, 10 µL *O*-methoxyamine hydrochloride (15 mg/mL) in pyridine was added to each sample and vortexed at 3200 rpm for 5 min, followed by incubation in the dark for 16 h. After this time, 10 µL of *N*,*O*-Bis(trimethylsilyl) trifluoroacetamide with 1% trimethylchlorosilane was added and incubated at 70 °C for 1 h. Finally, 50 µL of methyl stearate in heptane (C18:0, 10 mg/L) as an internal standard was added and vortexed for 10 min at 3200 rpm.

To acquire data, an Agilent Technologies 7890B GC system was used in combination with an Agilent Technologies 7250 QTOF mass spectrometer system. Derivatized samples (1 µL) were injected onto a HP-5MS (30 m, 0.25 mm, 0.25 µm; Agilent, USA) column in split mode (split ratio 30). The oven temperature was programmed from 60 °C (1 min) and increased at a rate of 10 °C/min until 325 °C (10 min). The transfer line temperature to the detector, the source filament temperature, and the quadrupole temperature were maintained at 280 °C, 230 °C, and 150 °C, respectively.

### 2.4. Lipidomic Analysis by RP-LC-QTOF-MS

A total of 100 µL of plasma was extracted with 350 µL of cold methanol (−20 °C) and 350 µL of methyl tert-butyl ether, followed by vortexing for 5 min. Then, samples were centrifuged at 13,000 rpm, 20 °C for 10 min. The samples were analyzed using the same LC-QTOF-MS system as described previously. A total of 5 µL of the obtained extract was injected into a C18 column (100 mm × 3.0 mm, 2.7 µm; Agilent, USA) at 40 °C with a gradient elution composed of 10 mM ammonium acetate H_2_O:MeOH (90:10) (Phase A) and 10 mM ammonium acetate ACN:MeOH:IPA (20:30:50) (Phase B) at a constant flow rate of 0.6 mL/min. The flow of B started at 70% and remained constant for 1 min. Afterward, it increased to 86% over the course of 2.5 min and remained at this level until reaching 10 min. Then, it increased to 100% for 1 min and was maintained for 7 min. Following this, it decreased for 3 min to recondition the equipment. Mass spectrometry detection was carried out in positive ESI mode throughout a full-scan range of 100 to 1100 *m*/*z*. The QTOF instrument was operated in 4 GHz (high-resolution) mode. For mass correction, two reference masses, *m/z* 121.0509 (purine, [C_5_H_4_N_4_+H]^+^) and *m/z* 922.0098 (HP-0921, [C_18_H_18_O_6_N_3_P_3_F_24_+H]^+^), were utilized throughout the analysis. MS/MS acquisition mode was performed by data-dependent acquisition mode, using a QC sample at different collision energies of 20 and 40 eV.

### 2.5. Quality Assurance (QA) and Quality Control Samples (QC)

For both LC and GC techniques, QA/QC procedures followed published guidelines to minimize undesired variability [17]. At the beginning of each sequence, system suitability procedures and tests were carried out to guarantee the appropriate instrumentation performance. Solvent blanks and extraction blanks were also analyzed to handle the undesirable and unavoidable signals from the materials and reagents used in the sample preparation. A pooled quality control sample (QC) was prepared by combining equal aliquots from each plasma sample, using the same procedure for both metabolomic and lipidomic analysis. This QC was injected ten times at the beginning of the run and after every ten samples. Additionally, to mitigate any potential bias, the biological samples were randomly arranged within the sequence.

### 2.6. Data Processing and Analysis

The raw data obtained from the LC-QTOF-MS system was processed using Agilent MassHunter Profinder B.10.0 software for deconvolution, alignment, and integration. In the case of GC-QTOF-MS data, the same processes were performed utilizing the following software tools: Agilent Unknowns Analysis B.10.0, MassProfiler Professional B.15.0, and Agilent Mass Hunter Quantitative Analysis B.10.0, respectively. The data matrix was normalized using the systematic error removal using random forest method server (https://slfan2013.github.io/SERRF-online/ (accessed on 5 May 2022)), based on the utilization of quality control pool samples [18]. Following that, data from all platforms were carefully examined. A presence and reproducibility filter were applied, and only the metabolites present in at least 80% of the samples within the same group and that demonstrated a coefficient of variation (CV, %) in the QC samples below 20% for LC data (30% for GC data) were considered for statistical analysis.

To identify the molecular features with statistically significant differences between groups, univariate (UVA) and multivariate (MVA) statistical analyses were employed. *p*-Values for UVA analysis were computed using nonparametric tests (Mann–Whitney U test) in Matlab. PCA was employed as an unsupervised method in MVA analysis to assess the quality of the data and the unsupervised samples distribution. Following that, supervised orthogonal partial least squares discriminant analysis (OPLS-DA) models were applied to determine the molecular attributes responsible for the group separation. The quality and performance of the multivariate OPLSDA models were assessed using the R^2^, Q^2^, permutation test, and cross-validation analysis of variance values. For MVA analysis, the SIMCA-P+16.0 program (Umetrics) was used. The statistically significant features chosen met at least one of the following requirements: (1) UVA—*p*-value < 0.05 and (2) MVA—variance important in projection (VIP) > 1.

### 2.7. Metabolites Identification

Multiple parameters were used to annotate significant features analyzed by liquid chromatography, including verification of retention times and probability of adduct formation, comparison of high-resolution masses with database records using the CEU Mass Mediator tool (http://ceumass.eps.uspceu.es (accessed on 10 May 2022)), and generation of theoretical formulas using isotopic distributions. MS/MS data were compared with the spectra data available in MS-DIAL 4.80 (http://prime.psc.riken.jp/compms/msdial/main.html (accessed on 31 May 2022)), the Lipid Annotator software v10.0, and the GNPS server. Manual interpretation of the MS/MS spectrum was also conducted. The identification of compounds through GC analysis was performed by comparing the mass spectrum and FAMES retention index with those reported in the Fiehn GC-MS Metabolomics RTL (Retention Time Locked) Library 2013 [19]. Finally, the identification levels were assigned for each platform according to Metabolomics Standards Initiative guidelines outlined by Blaženović, I. et al. [20].

### 2.8. Altered Metabolite Pathway Mapping and Identification of Potential Diagnostic Biomarkers for PCA Patients

The analysis of altered metabolic pathways in the experimental groups was performed using the “Pathway Analysis” tool available on the MetaboAnalyst 5.0 site (http://www.metaboanalyst.ca/ (accessed on 29 October 2022)). For it, the altered metabolites were compared to the Homo sapiens (KEGG) metabolome database, which was accessible on the same website. Finally, to identify potential diagnostic biomarkers for PCa patients, Prism 8.0.2 (GraphPad, La Jolla, CA, USA) software was utilized to generate Receiver Operating Characteristic (ROC) curves. These curves provide valuable insights into the diagnostic accuracy of the selected metabolic markers.

## 3. Results

### 3.1. Description of the Cohorts

The cohort included 108 Colombian men with an average age of 61 years and a similar range, except for the group of healthy volunteers (Table 1). Other anthropometric variables, such as body mass index (25.2 ± 3.18), height (1.70 ± 0.06 m), and weight (72.5 ± 10.41 kg), were consistent across the groups. PSA levels varied across the groups, with higher averages observed in patients with Met (247 ± 418 ng/mL), PCa (17.3 ± 15.0 ng/mL), and BPH (9.08 ± 4.30 ng/mL) compared to HV (0.558 ± 0.28 ng/mL).

### 3.2. Untargeted Metabolomic and Lipidomic Analyses

**Figure 1:**
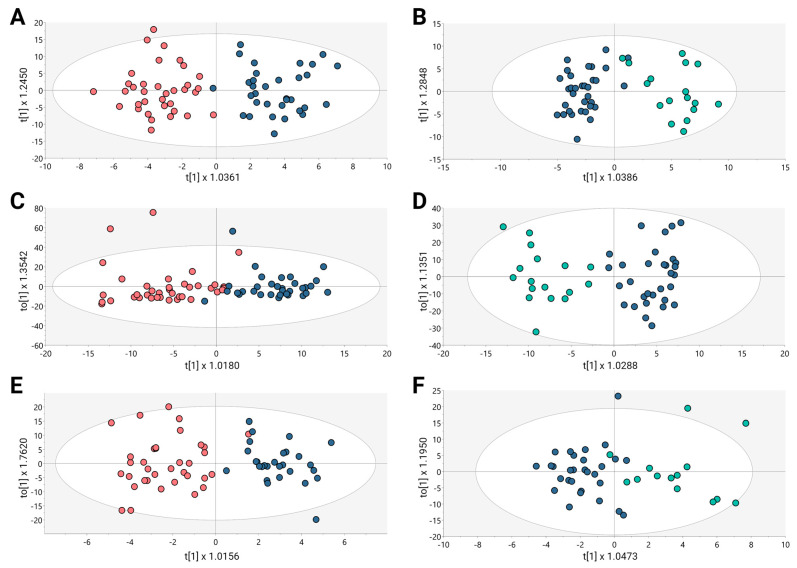
OPLS-DA models for metabolic and lipidomic analysis. (**A**,**C**,**E**) BPH vs. PCa, (**B**,**D**,**F**) Pca vs. MET. (**A**) GM(+):R^2^Y: 0.827, Q^2^: 0.231, CV-ANOVA: 0.011; (**B**) GM(+):R^2^Y: 0.829, Q^2^: 0.254, CV-ANOVA: 0.009; (**C**) GL(+):R^2^Y: 0.716, Q^2^: 0.331, CV-ANOVA: 0.004; (**D**) GL(+):R^2^Y: 0.852, Q^2^: 0.455, CV-ANOVA: 0.001; (**E**) GC: R^2^Y: 0.789, Q^2^: 0.36, CV-ANOVA: 0.012; (**F**) GC: R^2^Y: 0.697, Q^2^: 0.344, CV-ANOVA: 0.0031. Dots in salmon, blue, and cyan colors denote samples from patients with BPH, PCa, and Met, respectively.

Untargeted metabolomics analysis was conducted to evaluate the altered metabolomic profiles associated with BPH, PCa, and Met in a cohort of Colombian patients. A multiplatform strategy was implemented to identify the highest possible number of metabolites exhibiting alterations. The examination of the clusters revealed the grouping of quality control samples within the utilized analytical platforms (Figure S1, orange dots). After each analytical platform’s performance had been verified, the supervised orthogonal partial least squares regression approach (OPLS-DA) was used to improve the distinctions between the groups that included PCa and BPH (Figure 1A,C,E) and PCa and Met (Figure 1B,D,F). This approach was aimed at identifying the molecular characteristics that significantly influenced the segregation of these groups.

The OPLS-DA scoring plot in Figure 1 exhibited a distinct clustering between the groups: BPH (salmon dots), PCa (blue dots) and Met (cyan dots). Furthermore, the metrics R^2^ and Q^2^, which assess the model’s goodness of fit and predictive capacity, respectively, based on the data, yielded acceptable values. To assess the model’s reliability, cross-validation variance (cv-ANOVA) was conducted, confirming significant models across all analyzed platforms (cv-ANOVA < 0.05); this suggests that the multivariate models did not exhibit overfitting [20]. A total of 100 random permutation tests were conducted to investigate the potential overfitting of the supervised OPLS-DA models. The Y-intercepts of the Q^2^ distributions were consistently below zero across all implemented analytical platforms (Supplementary Figure S2), indicating the reliability of the established OPLS-DA model.

A combination of multivariate analysis (MVA) with a variable importance in projection (VIP) threshold of greater than 1 and univariate analysis (UVA) with a significance level (*p*-value) of less than 0.05 was employed to identify specific distinguishing metabolites. This approach resulted in the identification of a total of 104 altered metabolites covering all the platforms used in the study. Among these metabolites, 14.42% were found to be increased, while 85.58% decreased when comparing individuals with BPH to those with PCa. Additionally, 81 altered compounds were found in the comparison of PCa and MET patients, with 37.04% showing a trend downward and 62.96% showing a trend up-ward. A detailed list of the altered metabolites observed in BPH, PCa, and Met patients can be found in Supplementary Table S1.

**Figure 2:**
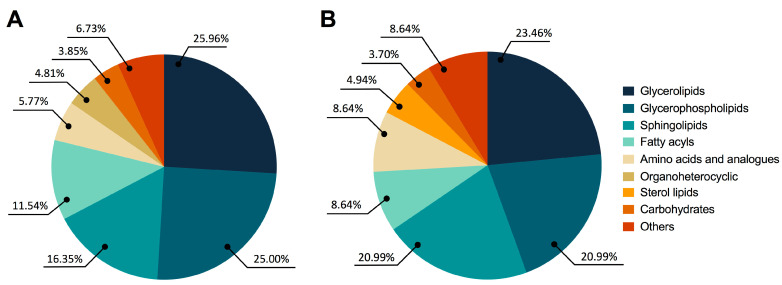
Altered metabolites among BPH, PCa, and MET patients. (**A**) BPH vs. PCa patients. (**B**) PCa vs. MET patients. The chemical classes are shown according to the color code.

Figure 2A shows significant global changes between BPH and PCa in the following categories: glycerolipids (25.96%), glycerophospholipids (25.00%), sphingolipids (16.35%), fatty acyls (11.54%), amino acids and analogues (5.77%), organoheterocyclic compounds (4.81%), carbohydrates (3.85%), and other compounds (6.73%). Similar patterns were seen in the fluctuations of glycerolipids (23.46%), glycerophospholipids (20.99%), sphingolipids (20.99%), fatty acyls (8.64%), amino acids and analogues (8.64%), sterol lipids (4.94%), carbohydrates (3.70%), and other compounds (8.64%) in PCa and Met patients, as shown in Figure 2B.

In comparison to BPH patients, PCa patients exhibited a preferential decrease in the altered metabolite trends for amino acids, glycerolipids, glycerophospholipids, sphingolipids, and carnitines. No discernible trend was observed for fatty acids or carbohydrates. Interestingly, metabolites involved in coenzyme A metabolism were found to be reduced in the PCa group. Furthermore, when compared to Met patients, PCa patients showed a decrease in metabolites from the glycerolipid, glycerophospholipid, and sphingolipid groups, along with an increase in amino acids and carbohydrates (Supplementary Table S1). In summary, PCa patients exhibited lower levels of glycerolipids, glycerophospholipids, and sphingolipids compared to both BPH and Met patients.

**Figure 3:**
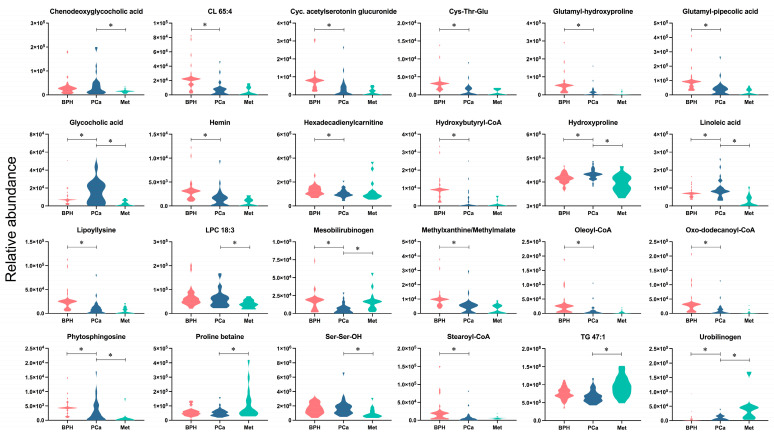
Altered metabolites among BPH, PCa, and Met patients. The colors salmon, blue, and light blue correspond to the BPH, PCa, and Met groups, respectively. * Denotes significant difference (*p* < 0.05) between the compared groups.

After analyzing the metabolites presented in Figure 3, classified according to the largest changes (FC < 0.5, FC > 1.5), the metabolites CL 65:4, cyclic acetylserotonin glucuronide, Cys-Thr-Glu, glutamyl-hydroxyproline, glutamyl-pipecolic acid, hemin, hexadecadienylcarnitine, hydroxybutyryl-CoA, lypollysine, methylxanthine/methyilmalate, oleoyl-CoA, oxo-dodecanoyl-CoA, and stearoyl-CoA were significantly altered between the BPH and PCa groups, with decreased levels observed in the latter group. Furthermore, the metabolites chenodeoxyglycocholic acid, LPC18:3, and Ser-Ser-OH showed significant increases, and TG 47:1 and proline betaine, which decreased, were significantly different from the PCa group compared to the Met group. Another group of compounds, including glycocholic acid, hydroxyproline, linoleic acid, mesobilirubinogen, phytosphingosine and urobilinogen, exhibited significant changes across the three groups. The relative abundances of substances like glycocholic acid, hydroxyproline, linoleic acid and phytosphingosine are interesting because they were found to only be enhanced in PCa patients. Additionally, the Met group was characterized by increased levels of urobilinogen, a metabolite that significantly differed from the BPH and PCa groups.

**Figure 4:**
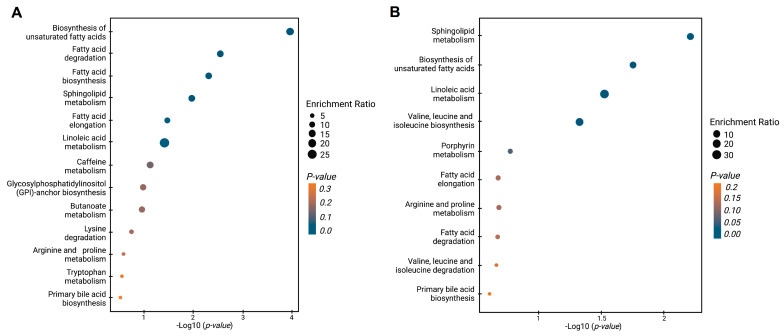
Enrichment analysis of altered pathways in the comparisons between BPH, PCa, and Met patients. (**A**) BPH vs. PCa patients. (**B**) PCa vs. MET patients. The significance of pathway alteration is indicated according to the color scale. Dots in yellow colors represent nonsignificant alterations (*p >* 0.05), and blue represents significant alterations (*p <* 0.05).

Figure 4 shows the enrichment analysis, which reveals the dysregulated metabolic pathways when BPH, PCa, and Met patients are contrasted. The comparison between PCa and BPH patients (Figure 4A) revealed multiple dysregulated pathways (highlighted in various shades of blue), primarily related to lipid metabolism. These pathways include the biosynthesis of unsaturated fatty acids, fatty acid degradation and elongation, sphingolipid metabolism, and linoleic acid metabolism. Other pathways also showed some level of impact but with lesser significance. The comparison of PCa and Met patients also revealed significant changes in lipid-related pathways, in line with the previous results. Among the most significant pathways were those involved in the biosynthesis of unsaturated fatty acids, sphingolipid metabolism, and linoleic acid metabolism. The biosynthetic pathway for valine, leucine, and isoleucine exhibited significant alterations. In summary, the global trends, along with the analysis of dysregulated pathways in PCa vs. BPH and PCa vs. Met comparisons are primarily changes related to the lipid profile, which suggests that patients with PCa generally have lower levels of lipids compared to patients with BPH and Met.

### 3.3. Potential Diagnostic Biomarkers among BPH and PCa Patients

**Figure 5:**
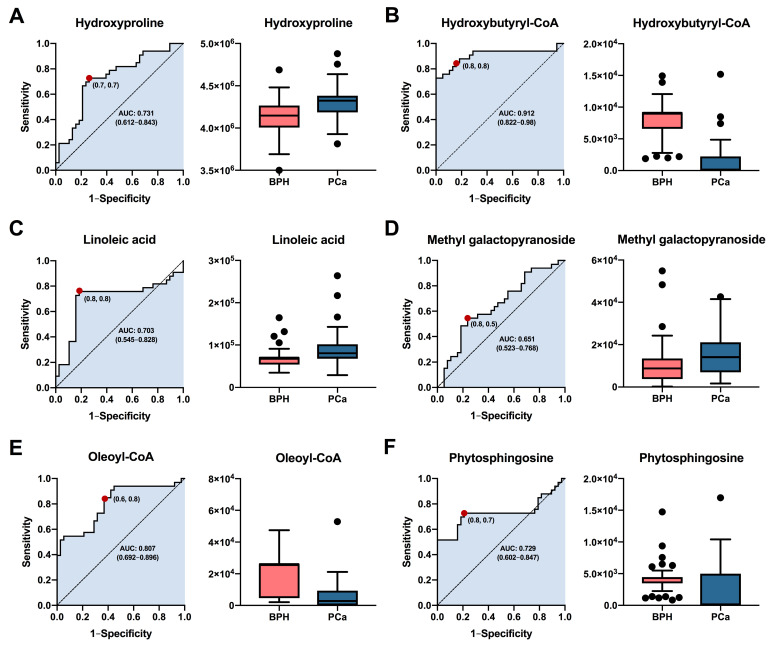
Analysis of the receiver operating characteristics of potential plasma biomarkers for diagnostic between patients with PCa and BPH. ROC curve analysis and box plot of metabolites with the highest contribution of separating the studied groups. (**A**) Hydroxyproline, (**B**) Hydroxybutyryl-CoA, (**C**) Linoleic acid, (**D**) Methyl galactopyranoside, (**E**) Oleoyl-CoA, (**F**) Phytosphingosine. Box plot in salmon and blue colors, representing BPH and PCa patients, respectively. Data are shown by the median, with the range from minimum to maximum.

Based on their highest levels of identification (ID level 1-2), fold change, VIP values, and *p*-values with FDR, a total of six metabolites (hydroxyproline, hydroxybutyryl-CoA, linoleic acid, methyl galactopyranoside, oleoyl-CoA, and phytosphingosine) were selected for potential involvement in the prognostic between BPH and PCa groups. The predictive capacity of each selected biomarker, measured through ROC analysis, is depicted in Figure 5. Interestingly, the results showed that hydroxybutyryl-CoA (Figure 5B) exhibited the highest performance with area under the curve (AUC) values of 0.912 (CI: 0.8222–0.98), along with selectivities and sensitivities of 0.8. Other compounds such as hydroxyproline (Figure 5A), linoleic acid (Figure 5C), oleoyl-CoA (Figure 5E), and phytospingosine (Figure 5F) demonstrated acceptable AUC values of 0.731 (CI: 0.612–0.843), 0.703 (CI: 0.545–0.828), 0.807 (CI: 0.692–0.896), and 0.729 (CI: 0.602–0.847), respectively. On the other hand, methyl galactopyranoside (Figure 5D) exhibited poor performance, with AUC values below 0.7. In summary, the results obtained suggest that metabolites such as hydroxybutyryl-CoA, oleoyl-CoA, hydroxyproline, and phytosphingosine could be used as prognostic biomarkers for both BPH and PCa patients. It is essential that future studies assess the performance of these potential biomarkers in diverse patient cohorts.

### 3.4. Subgrouping of the Samples Belonging to the PCa Group

Different methodologies were employed to cluster the samples belonging to this group to establish probable metabolomic profiles among subclassifications within the PCa patient group. The Gleason score system, which is based on microscopic examination of cancer cells during a biopsy or prostatectomy, PSA levels, and the TNM (tumor, nodes, metastasis) staging system, which is used to describe the degree and spread of prostate cancer, were all examined. The Gleason scoring system provides a grade from 1 to 5 to the most prevalent spots of cancer cells, and the sum of the two most common grades yields a final score. The samples were grouped as follows: total Gleason score 6 (3 + 3), 7 (3 + 4/4 + 3), 8 (4 + 4), 9 (4 + 5/5 + 4). Regarding the subgroups based on PSA level, the samples were classified as follows: low (≤10), intermediate (10–20), high (≥20). TNM staging is based on three major factors: the size of the primary tumor, the presence of affected lymph nodes, and the occurrence of metastasis in other organs. Each component is assigned a numerical value, which is then added together to define the cancer stage (T0, T1, T2). However, in both multivariate and univariate models, employing these subgroupings did not result in precise clustering of the samples (Supplementary Figure S3), displaying high heterogeneity among PCa patient samples.

## 4. Discussion

Prostate cancer is a highly heterogeneous tumor with a wide range of clinical outcomes, ranging from innocuous indolent tumors to aggressive metastasizing tumors [21]. It has a wide range of morphological patterns as well as some unusual metaplastic differentiation [22]. The vast variability of prostate cancer is still insufficiently understood. Nevertheless, spatial heterogeneity and other forms of genetic and molecular heterogeneity, including interpatient, intertumoral, and intratumoral variability, play important roles in disease pathogenesis [22,23,24,25]. Others factors, including disparities in PSA testing practices among nations [26,27], in conjunction with occidental dietary patterns, sedentary lifestyles and obesity [27], tumor microenvironment [28], differing healthcare systems, population longevity, and other mortality causes collectively contribute to a multifaceted scenario [27]. These factors impact global prostate cancer incidence and mortality rates, presenting challenges to developing suitable therapies and exploring alternative diagnostic and prognostic strategies.

**Figure 6:**
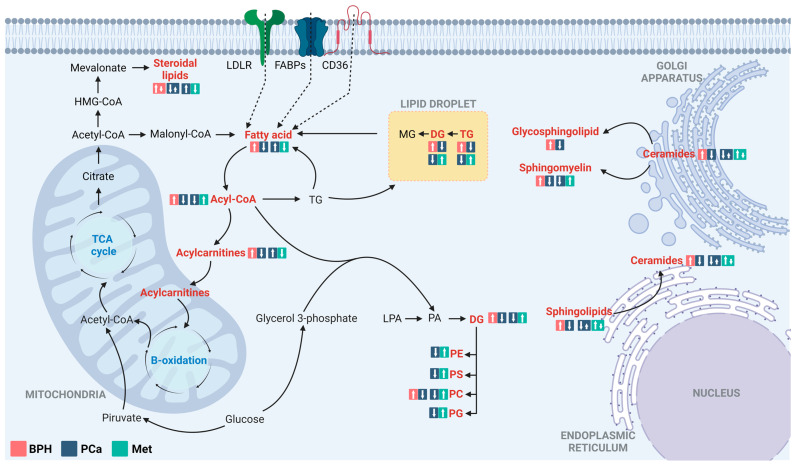
Altered lipid metabolism in comparisons between patients with BPH, PCa, and Met. The figure highlights the main alterations observed in comparisons of the BPH vs. PCa and PCa vs. Met groups, classified by compound families. The altered families are highlighted in red. The box in the salmon, blue, and light blue colors corresponds to the tendencies in BPH, PCa, and Met groups, respectively. LDLR: Low-density lipoprotein receptor; FSBPs: Fatty acid binding proteins; CD36: N-linked glycosylated transmembrane protein; MG: Monoacylglycerols; DG: Diacylglycerol; TG: Triacylglycerol; PC: Glycerophosphocholine, PE: Glycerophosphoethanolamine; PS: Glycerophosphoserine; PG: Glycerophosphoglycerols; PA: Phosphatidic acids; LPA: Lysophosphatidic acids. Adapted from [30].

For better comprehension of the underlying physiological processes of the disease, chromatographic techniques coupled with high-resolution mass spectrometry have been employed to characterize altered metabolic profiles in Colombian patients with BPH, PCa, and Met. According to the findings, the predominant metabolic changes between BPH, PCa, and Met patients are related to lipid metabolism (Figure 6). Lipids are essential for tumor growth and metastasis, and they play a significant role in malignant tumors. In addition to their role as components of cell membranes, lipids serve multiple functions in cancer cells. They act as a significant energy source [29,30,31], functioning as crucial signaling molecules [32,33]. Additionally, they influence the tumor microenvironment by promoting events including inflammation, angiogenesis, and immune suppression [31,33], and they also contribute to altering membrane composition [33]. 

Throughout the obtained results, we observed that both PCa and BPH patients exhibit elevated biosynthetic pathways associated with lipids, including biosynthesis of unsaturated fatty acids, fatty acid degradation and elongation, sphingolipid metabolism, and linoleic acid metabolism, compared to healthy volunteers. These results are consistent with observations from other studies where it has been reported that this particular category of compounds in PCa is increasing [34,35,36]. This increase is linked to the androgen receptor, which regulates lipid metabolism, particularly by inducing the expression of sterol regulatory element-binding and fatty acid synthase proteins [37]. Similarly, the severity of inflammation in the prostatectomy sample has also demonstrated a positive correlation with hyperlipidemia, and prostate size has also been found to be strongly connected to the degree of prostatic inflammation [38,39]. Collectively, these findings imply that hyperlipidemia could potentially play a pivotal role in both BPH and PCa. The relationship between BPH and PCa remains a matter of debate [40,41,42,43,44]. Nevertheless, indications have arisen that suggest an increased incidence of PCa in Korean patients with BPH and/or prostatitis. Notably, this association was most pronounced in cases where both BPH and prostatitis were present [41]. Similarly, a study involving Danish men observed a two- to three-fold increased risk of PCa incidence and a two- to eight-fold increased risk of PCa mortality in individuals with clinical BPH during a follow-up period of up to 27 years [44]. 

BPH and PCa are linked by a variety of variables, including metabolic syndrome, hormones, and inflammation. Notably, inflammatory mediators may contribute to PCa through a variety of signaling pathways, including apoptosis suppression, cell growth stimulation, and tumor suppressor gene loss [45]. The role of hyperlipidemia in the development of inflammation is particularly important. Furthermore, the findings suggest that lipid imbalance, and, hence, inflammatory induction, is more pronounced in BPH, reflecting a progressive development toward PCa. This suggests that early activation of lipid metabolism is crucial for cancer development and establishment [46]. On the other hand, metabolic changes play a pivotal role in tumor development and dissemination. While glucose is typically recognized as the primary metabolic substrate in rapidly growing tumors, other substrates, such as amino acids, pyruvate, lactate, and lipids, can also expedite the metastatic progression [46,47]. Lipid metabolism holds particular significance within the metastatic cascade, evident in elevated de novo lipid synthesis via ATP-citrate lyase and fatty acid synthase, along with heightened expression of fatty acid transporters (CD36). The accumulation of lipids in lipid droplets has been identified as a means to energetically support invasiveness [48]. Moreover, membrane lipid imbalances may foster invasion and migration processes triggered by the epithelial–mesenchymal transition of metastasis-initiating cells, further facilitating the extravasation of cancer cells from the bloodstream into distant organs [46,47]. 

As previously described, there has generally been an observed positive correlation between lipid levels and metastasis progression. However, this topic remains controversial and is currently a focal point within the scientific community. Some research suggests the possibility of a negative regulation of lipid metabolism. For instance, a study that combined the proteogenomic features of metastatic colorectal cancer tumors found that proteins enriched in metabolic pathways, such as fatty acid degradation, citrate cycle, and oxidative phosphorylation, were downregulated in metastatic tumors [49]. In the same way, the role of the fatty acid transporter CD36 has been assessed in pancreatic tumor cells, a cancer type known for its significant invasion and metastatic potential. The CD36 gene has been found to express itself at low levels in pancreatic cancer. Reduced CD36 expression is associated with bigger tumor sizes and a poor prognosis for survival [50]. Interestingly, the metabolic profiles of Met and PCa, analyzed in this research, revealed lower levels of fatty acids, carnitines, and steroid lipids, along with higher levels of sphingolipids, glycerophospholipids, and glycerolipids in Met patients compared to PCa patients. This may reflect the cancer’s heterogeneity, as the altered behavior of lipidic families during the PCa–Met transition reveals multiple nuances that are not yet fully understood.

Considering the multiple functions performed by lipids in cellular homeostasis, it is intriguing to believe that dysregulation of this network plays a role in the development of many illnesses [51,52]. This characteristic has driven the search and development of lipids with potential in the diagnosis and prognosis of multiple diseases, particularly in cancer. For example, a prospective pilot study that included 74 PCa patients, 74 BPH patients, and 72 healthy subjects analyzed 18 lipid metabolites as potential biomarkers for the diagnosis of PCa with PSA levels in the gray zone of 4–10 ng/mL. This study found areas under the curve greater than 0.8 and sensitivities and specificities ranging from 71.62% to 93.24%. Interestingly, the trends of this group of lipid metabolites were found to be lower in the PCa group compared to the BPH group, which is consistent with the findings in this research [53]. Other lipids, such as nonanedioic acid, LPC 18:0, LPE 18:2, pregnanetriol glucuronide, decanoic acid (capric acid), heptadecanoic acid, and hexadecanedioic acid, have been described as potential diagnostic biomarkers distinguishing between PCa patients and healthy individuals [54]. 

In de novo lipid synthesis, acetyl-CoA serves as the fundamental building block for fatty acids and can be produced from citrate or acetate. This process is intricately linked to energy production. Numerous studies have identified disruptions in the processes related to acyl-CoA catabolism in a wide range of cancers, including colorectal, breast, melanoma, liver, lung, blood, and prostate cancers [55]. Given the involvement of acyl-CoA metabolism in the context of cancer, some research has considered it a potential diagnostic and prognostic biomarker. The enzyme long-chain fatty acyl-CoA synthetase, responsible for activating fatty acids by catalyzing the condensation of fatty acids with a molecule of coenzyme A to form a thioester, has been shown through a meta-analysis of publicly available gene expression databases to exhibit a positive correlation with a distinct subtype of triple negative breast cancer. This subtype is characterized by the absence of the androgen receptor and is associated with an aggressive breast cancer phenotype [56]. Similarly, the enzyme acetyl coenzyme A synthase 2, responsible for converting acetate to acetyl coenzyme A, was found to be upregulated in cervical squamous cell carcinoma tissues through data mining and in vitro experiments. This discovery revealed that acetyl coenzyme A synthase 2 is associated with a poor prognosis in this context [57]. The present research identified metabolites such as hydroxybutyryl-CoA and oleoyl-CoA, which exhibited AUC values greater than 0.8 and could be associated with the diagnostic of BPH to PCa. However, it is essential for this information to be validated in a new cohort study.

The main limitation of the present investigation involves the age difference between the healthy controls and the other groups. However, the study’s purpose only considered comparisons between BPH, PCa, and Met. A new group will be required to validate the potential biomarkers provided as well as to implement guided procedures for their quantification. In the future, we intend to apply modifications to these parameters to obtain more robust data through semi-targeted and quantitative lipidomics studies.

## 5. Conclusions

The present study established the metabolic features of BPH, PCa, and Met in plasma metabolites using LC-QTOF-MS and GC-QTOF-MS and provided evidence of changes in chemical families such as fatty acids, glycerolipids, glycerophospholipids, and sphingolipids throughout PCa development. In addition, we also address how the lipid imbalance in patients with BPH, PCa, and Met changes according to each of the diseases. This shows that the dynamics of lipid family levels may be shifting, thereby participating in the development and establishment of each pathology. Future studies should take this into consideration.

## Figures and Tables

**Table 1:** Clinical variables evaluated in the HV, BPH, PCa, and Met individual cohorts.

Variables	HV	BPH	PCa	Met	Overall
	(*N* = 20)	(*N* = 37)	(*N* = 34)	(*N* = 17)	(*N* = 108)
Age (years)					
Mean (SD)	29.7 (3.20)	66.4 (6.50)	67.2 (7.81)	73.0 (8.54)	60.9 (16.60)
Median [Min, Max]	29.0 [25.0, 34.0]	67.0 [55.0, 80.0]	68.0 [52.0, 82.0]	74.0 [61.0, 88.0]	64.0 [25.0, 88.0]
Anthropometric measurement					
Weight (kg)					
Mean (SD)	79.6 (7.58)	71.9 (9.63)	73.6 (10.3)	63.2 (8.52)	72.5 (10.4)
Median [min, max]	79.0 [65.0, 98.0]	70.0 [49.0, 100]	72.5 [48.0, 94.0]	63.0 [42.0, 78.0]	71.9 [42.0, 100]
Height (m)					
Mean (SD)	1.75 (0.05)	1.68 (0.06)	1.69 (0.05)	1.67 (0.06)	1.70 (0.06)
Median [min, max]	1.76 [1.65, 1.85]	1.68 [1.56, 1.80]	1.69 [1.53, 1.77]	1.67 [1.57, 1.80]	1.70 [1.53, 1.85]
Body Mass Index					
Mean (SD)	26.0 (2.79)	25.4 (2.82)	25.9 (3.46)	22.6 (2.58)	25.2 (3.18)
Median [min, max]	25.5 [22.5, 33.9]	25.4 [16.8, 33.0]	25.6 [19.2, 34.7]	22.5 [16.0, 28.7]	25.1 [16.0, 34.7]
Prostate-Specific Antigen (ng/mL)					
Mean (SD)	0.558 (0.28)	9.08 (4.30)	17.3 (15.0)	247 (418)	43.8 (174)
Median [min, max]	0.470 [0.250, 1.2]	8.00 [3.10, 22.5]	11.7 [1.09, 64.0]	64.0 [0.15, 1590]	8.05 [0.15, 1590]
Missing	0 (0%)	0 (0%)	0 (0%)	2 (11.8%)	2 (1.9%)

HV: healthy volunteers, BPH: benign prostate hyperplasia, PCa: prostate cancer, Met: metastasis.

## Data Availability

The data presented in this study are available in this article.

## Supplementary Materials

The following supporting information can be downloaded at https://www.mdpi.com/article/10.3390/cancers15225465/s1, Supplementary Table S1: Altered metabolites between PCa vs. BPH and PCa vs. Met comparisons; Figure S1: PCA models for metabolic and lipidomic analysis; Figure S2: OPLS-DA permutation test plots; Figure S3: PLSDA Models for comparisons regarding PSA, TNM, and Gleason score classifications.

## References

[1] Global Cancer Observatory. https://gco.iarc.fr/today.

[2] Sharma S., Zapatero-Rodríguez J., O’Kennedy R. (2017). Prostate Cancer Diagnostics: Clinical Challenges and the Ongoing Need for Disruptive and Effective Diagnostic Tools. Biotechnol. Adv..

[3] De Koning H.J., Auvinen A., Sanchez A.B., Da Silva F.C., Ciatto S., Denis L., Gohagan J.K., Hakama M., Hugosson J., Kranse R. (2002). Large-Scale Randomized Prostate Cancer Screening Trials: Program Performances in the European Randomized Screening for Prostate Cancer Trial and the Prostate, Lung, Colorectal and Ovary Cancer Trial. Int. J. Cancer.

[4] Bangma C.H., Roemeling S., Schröder F.H. (2007). Overdiagnosis and Overtreatment of Early Detected Prostate Cancer. World J. Urol..

[5] DeSantis C.E., Lin C.C., Mariotto A.B., Siegel R.L., Stein K.D., Kramer J.L., Alteri R., Robbins A.S., Jemal A. (2014). Cancer Treatment and Survivorship Statistics, 2014. CA Cancer J. Clin..

[6] Vander Heiden M.G., DeBerardinis R.J. (2017). Understanding the Intersections between Metabolism and Cancer Biology. Cell.

[7] Faulds M.H., Dahlman-Wright K. (2012). Metabolic Diseases and Cancer Risk. Curr. Opin. Oncol..

[8] Peng G., Pakstis A.J., Gandotra N., Cowan T.M., Zhao H., Kidd K.K., Scharfe C. (2022). Metabolic Diversity in Human Populations and Correlation with Genetic and Ancestral Geographic Distances. Mol. Genet. Metab..

[9] Mullins J.K., Loeb S. (2012). Environmental Exposures and Prostate Cancer. Urol. Oncol. Semin. Orig. Investig..

[10] Hinata N., Fujisawa M. (2022). Racial Differences in Prostate Cancer Characteristics and Cancer-Specific Mortality: An Overview. World J. Men’s Health.

[11] Sciacovelli M., Gaude E., Hilvo M., Frezza C. (2014). The Metabolic Alterations of Cancer Cells. Methods in Enzymology.

[12] Oermann E.K., Wu J., Guan K.L., Xiong Y. (2012). Alterations of Metabolic Genes and Metabolites in Cancer. Semin Cell Dev. Biol..

[13] Suri G.S., Kaur G., Carbone G.M., Shinde D. (2023). Metabolomics in Oncology. Cancer Rep..

[14] Subramani R., Poudel S., Smith K.D., Estrada A., Lakshmanaswamy R. (2022). Metabolomics of Breast Cancer: A Review. Metabolites.

[15] Kdadra M., Höckner S., Leung H., Kremer W., Schiffer E. (2019). Metabolomics Biomarkers of Prostate Cancer: A Systematic Review. Diagnostics.

[16] (2023). EAU Guidelines. http://uroweb.org/guidelines/compilations-of-all-guidelines/.

[17] Kirwan J.A., Gika H., Beger R.D., Bearden D., Dunn W.B., Goodacre R., Theodoridis G., Witting M., Yu L.R., Wilson I.D. (2022). Quality Assurance and Quality Control Reporting in Untargeted Metabolic Phenotyping: MQACC Recommendations for Analytical Quality Management. Metabolomics.

[18] Fan S., Kind T., Cajka T., Hazen S.L., Tang W.H.W., Kaddurah-Daouk R., Irvin M.R., Arnett D.K., Barupal D.K., Fiehn O. (2019). Systematic Error Removal Using Random Forest for Normalizing Large-Scale Untargeted Lipidomics Data. Anal. Chem..

[19] Kind T., Wohlgemuth G., Lee D.Y., Lu Y., Palazoglu M., Shahbaz S., Fiehn O. (2009). FiehnLib: Mass Spectral and Retention Index Libraries for Metabolomics Based on Quadrupole and Time-of-Flight Gas Chromatography/Mass Spectrometry. Anal. Chem..

[20] Blaženović I., Kind T., Ji J., Fiehn O. (2018). Software Tools and Approaches for Compound Identification of LC-MS/MS Data in Metabolomics. Metabolites.

[21] Stattin P., Holmberg E., Johansson J.E., Holmberg L., Adolfsson J., Hugosson J. (2010). Outcomes in Localized Prostate Cancer: National Prostate Cancer Register of Sweden Follow-up Study. J. Natl. Cancer Inst..

[22] Tolkach Y., Kristiansen G. (2018). The Heterogeneity of Prostate Cancer: A Practical Approach. Pathobiology.

[23] Scattoni V., Maccagnano C., Capitanio U., Gallina A., Briganti A., Montorsi F. (2014). Random Biopsy: When, How Many and Where to Take the Cores?. World J. Urol..

[24] Gerlinger M., Catto J.W., Orntoft T.F., Real F.X., Zwarthoff E.C., Swanton C. (2015). Intratumour Heterogeneity in Urologic Cancers: From Molecular Evidence to Clinical Implications. Eur. Urol..

[25] Kumar A., Coleman I., Morrissey C., Zhang X., True L.D., Gulati R., Etzioni R., Bolouri H., Montgomery B., White T. (2016). Substantial Interindividual and Limited Intraindividual Genomic Diversity among Tumors from Men with Metastatic Prostate Cancer. Nat. Med..

[26] Dasgupta P., Baade P.D., Aitken J.F., Ralph N., Chambers S.K., Dunn J. (2019). Geographical Variations in Prostate Cancer Outcomes: A Systematic Review of International Evidence. Front. Oncol..

[27] Center M.M., Jemal A., Lortet-Tieulent J., Ward E., Ferlay J., Brawley O., Bray F. (2012). International Variation in Prostate Cancer Incidence and Mortality Rates. Eur. Urol..

[28] Ge R., Wang Z., Cheng L. (2022). Tumor Microenvironment Heterogeneity an Important Mediator of Prostate Cancer Progression and Therapeutic Resistance. NPJ Precis Oncol..

[29] Deberardinis R.J., Thompson C.B. (2012). Cellular Metabolism and Disease: What Do Metabolic Outliers Teach Us?. Cell.

[30] Fu Y., Zou T., Shen X., Nelson P.J., Li J., Wu C., Yang J., Zheng Y., Bruns C., Zhao Y. (2021). Lipid Metabolism in Cancer Progression and Therapeutic Strategies. MedComm.

[31] Bian X., Liu R., Meng Y., Xing D., Xu D., Lu Z. (2021). Lipid Metabolism and Cancer. J. Exp. Med..

[32] Luo X., Zhao X., Cheng C., Li N., Liu Y., Cao Y. (2018). The Implications of Signaling Lipids in Cancer Metastasis. Exp. Mol. Med..

[33] Vasseur S., Guillaumond F. (2022). Lipids in Cancer: A Global View of the Contribution of Lipid Pathways to Metastatic Formation and Treatment Resistance. Oncogenesis.

[34] Crowe F.L., Allen N.E., Appleby P.N., Overvad K., Aardestrup I.V., Johnsen N.F., Tjønneland A., Linseisen J., Kaaks R., Boeing H. (2008). Fatty Acid Composition of Plasma Phospholipids and Risk of Prostate Cancer in a Case-Control Analysis Nested within the European Prospective Investigation into Cancer and Nutrition. Am. J. Clin. Nutr..

[35] Zhou X., Mao J., Ai J., Deng Y., Roth M.R., Pound C., Henegar J., Welti R., Bigler S.A. (2012). Identification of Plasma Lipid Biomarkers for Prostate Cancer by Lipidomics and Bioinformatics. PLoS ONE.

[36] Freeman V.L., Flanigan R.C., Meydani M. (2007). Prostatic Fatty Acids and Cancer Recurrence after Radical Prostatectomy for Early-Stage Prostate Cancer. Cancer Causes Control.

[37] Siltari A., Syvälä H., Lou Y.R., Gao Y., Murtola T.J. (2022). Role of Lipids and Lipid Metabolism in Prostate Cancer Progression and the Tumor’s Immune Environment. Cancers.

[38] Vignozzi L., Gacci M., Cellai I., Santi R., Corona G., Morelli A., Rastrelli G., Comeglio P., Sebastanelli A., Maneschi E. (2013). Fat Boosts, While Androgen Receptor Activation Counteracts, BPH-Associated Prostate Inflammation. Prostate.

[39] Zhu C., Wu J., Wu Y., Guo W., Lu J., Zhu W., Li X., Xu N., Zhang Q. (2022). Triglyceride to High-Density Lipoprotein Cholesterol Ratio and Total Cholesterol to High-Density Lipoprotein Cholesterol Ratio and Risk of Benign Prostatic Hyperplasia in Chinese Male Subjects. Front. Nutr..

[40] Chokkalingam A.P., Nyrén O., Johansson J.E., Gridley G., McLaughlin J.K., Adami H.O., Hsing A.W. (2003). Prostate Carcinoma Risk Subsequent to Diagnosis of Benign Prostatic Hyperplasia: A Population-Based Cohort Study in Sweden. Cancer.

[41] Kim S.H., Kwon W.A., Joung J.Y. (2020). Impact of Benign Prostatic Hyperplasia and/or Prostatitis on the Risk of Prostate Cancer in Korean Patients. World J. Men’s Health.

[42] Schenk J.M., Kristal A.R., Arnold K.B., Tangen C.M., Neuhouser M.L., Lin D.W., White E., Thompson I.M. (2011). Association of Symptomatic Benign Prostatic Hyperplasia and Prostate Cancer: Results from the Prostate Cancer Prevention Trial. Am. J. Epidemiol..

[43] Ørsted D.D., Bojesen S.E. (2013). The Link between Benign Prostatic Hyperplasia and Prostate Cancer. Nat. Rev. Urol..

[44] Ørsted D.D., Bojesen S.E., Nielsen S.F., Nordestgaard B.G. (2011). Association of Clinical Benign Prostate Hyperplasia with Prostate Cancer Incidence and Mortality Revisited: A Nationwide Cohort Study of 3,009,258 Men. Eur. Urol..

[45] Sfanos K.S., de Marzo A.M. (2012). Prostate Cancer and Inflammation: The Evidence. Histopathology.

[46] Martin-Perez M., Urdiroz-Urricelqui U., Bigas C., Benitah S.A. (2021). Lipid Metabolism in Metastasis and Therapy. Curr. Opin. Syst. Biol..

[47] Bergers G., Fendt S.M. (2021). The Metabolism of Cancer Cells during Metastasis. Nat. Rev. Cancer.

[48] Corbet C., Bastien E., Santiago de Jesus J.P., Dierge E., Martherus R., Vander Linden C., Doix B., Degavre C., Guilbaud C., Petit L. (2020). TGFβ2-Induced Formation of Lipid Droplets Supports Acidosis-Driven EMT and the Metastatic Spreading of Cancer Cells. Nat. Commun..

[49] Li C., Sun Y.D., Yu G.Y., Cui J.R., Lou Z., Zhang H., Huang Y., Bai C.G., Deng L.L., Liu P. (2020). Integrated Omics of Metastatic Colorectal Cancer. Cancer Cell.

[50] Jia S., Zhou L., Shen T., Zhou S., Ding G., Cao L. (2018). Down-Expression of CD36 in Pancreatic Adenocarcinoma and Its Correlation with Clinicopathological Features and Prognosis. J. Cancer.

[51] Yan F., Zhao H., Zeng Y. (2018). Lipidomics: A Promising Cancer Biomarker. Clin. Transl. Med..

[52] Wenk M.R. (2005). The Emerging Field of lipidomics. Nat. Rev. Drug Discov..

[53] Xu B., Chen Y., Chen X., Gan L., Zhang Y., Feng J., Yu L. (2021). Metabolomics Profiling Discriminates Prostate Cancer from Benign Prostatic Hyperplasia within the Prostate-Specific Antigen Gray Zone. Front. Oncol..

[54] Zhang X., Xia B., Zheng H., Ning J., Zhu Y., Shao X., Liu B., Dong B., Gao H. (2022). Identification of Characteristic Metabolic Panels for Different Stages of Prostate Cancer by 1H NMR-Based Metabolomics Analysis. J. Transl. Med..

[55] Sebastiano M.R., Konstantinidou G. (2019). Targeting Long Chain Acyl-Coa Synthetases for Cancer Therapy. Int. J. Mol. Sci..

[56] Wu X., Li Y., Wang J., Wen X., Marcus M.T., Daniels G., Zhang D.Y., Ye F., Wang L.H., Du X. (2013). Long Chain Fatty Acyl-CoA Synthetase 4 Is a Biomarker for and Mediator of Hormone Resistance in Human Breast Cancer. PLoS ONE.

[57] Li C.J., Chiu Y.H., Chang C., Chang Y.C.I., Sheu J.J.C., Chiang A.J. (2021). Acetyl Coenzyme a Synthase 2 Acts as a Prognostic Biomarker Associated with Immune Infiltration in Cervical Squamous Cell Carcinoma. Cancers.

