# Supplementary material for: Unveiling Disrupted Lipid Metabolism in Benign Prostate Hyperplasia, Prostate Cancer, and Metastatic Patients: Insights from a Colombian Nested Case–Control Study
Source: Cancers (Basel). 2023 Nov 18;15(22):5465. doi: 10.3390/cancers15225465 (PMC10670336; doi:10.3390/cancers15225465)
Supplement: Supplementary file 1 [file cancers-15-05465-s001.zip › cancers-2670498-supplementary.pdf]

Supplementary Table S1. Altered metabolites between PCa vs BPH and PCa vs Met comparisons.

									PCa VS BPH			PCa VS Met		
**Compound**	**Formula**	**Mass**	**RT (min)**	**Error (ppm)**	**Adduct**	**[a]CV for QC (%)**	**Analytical platform**	**[b]ID level**	**[c]Fold Change**	**[d]*p* valueǂ**	**[e]VIP**	**[b]Fold Change**	**[d]*p* valueǂ**	**[e]VIP**
Amino acids and analogues														
Hydroxyproline	$C_5H_9NO_3$	131.0583	0.75	4	$[M+H]^+$	2.11	GM-LC-QTOF-MS	2	1.07	5.38E-02ǂ	1.71	1.10	6.09E-03ǂ	2.58
Methylene-glutamate	$C_6H_9NO_4$	159.0533	0.75	4	$[M+H]^+$	2.28	GM-LC-QTOF-MS	2	1.06		1.60	1.10	1.02E-02ǂ	2.58
Proline betaine	$C_7H_{13}NO_2$	125.0840	1.83	3	$[M+H-H_2O]^+$	8.65	GM-LC-QTOF-MS	4	--	--	--	0.49	--	1.66
Cysteine	$C_3H_7NO_2S$	121.197	13.62	-	-	12.96	GC-QTOF-MS	1	--	--	--	1.28	--	1.62
Valine	$C_5H_{11}NO_2$	117.1463	9.11	-	-	3.43	GC-QTOF-MS	1	--	--	--	1.25	3.23E-02ǂ	--
Cys Thr Glu	$C_{12}H_{21}N_3O_7S$	373.093	3.43	4	$[M+Na]^+$	6.97	GL-LC-QTOF-MS	4	0.32	8.60E-06	1.81	--	--	--
Glutamyl-pipecolic acid	$C_{11}H_{18}N_2O_5$	296.0755	5.74	6	$[M+K]^+$	4.24	GL-LC-QTOF-MS	3	0.39	1.87E-05	1.75	--	--	--
Glutamyl-hydroxyproline	$C_{10}H_{16}N_2O_6$	298.0546	4.98	6	$[M+K]^+$	4.18	GL-LC-QTOF-MS	4	0.28	6.62E-06	1.56	--	--	--
Methylene-glutamate/Methyl-oxoglutaramic acid/Methylnicotinamide	$C_6H_9NO_4$/$C_7H_9N_2O$	159.053	0.68	2	$[M+H]^+$/$[M+Na]^+$	1.61	GL-LC-QTOF-MS	3	--	--	--	1.10	--	1.50
Ser-Ser-OH	$C_{12}H_{14}N_2O_8$	314.0723	0.98	7	$[M+H]^+$	4.11	GL-LC-QTOF-MS	3	--	--	--	1.80	3.66E-02	1.66
Carbohydrates														
Gluconic acid	$C_6H_{12}O_7$	196.1553	17.49	-	-	8.86	GC-QTOF-MS	1	1.47	3.19E-02	--	--	--	--
Methyl galactopyranoside	$C_7H_{14}O_6$	194.1825	16.61	-	-	7.68	GC-QTOF-MS	1	1.41	3.48E-02ǂ	--	--	--	--
Palmityl-CoA	$C_37H_{66}N_7O_{17}P_3S$	1043.306	8.46	6	$[M+K]^+$	6.27	GL-LC-QTOF-MS	4	0.57	1.27E-03	1.19	--	--	--
Cyclic acetylserotonin glucuronide	$C_{18}H_{20}N_2O_8$	430.0797	5.16	6	$[M+K]^+$	4.51	GL-LC-QTOF-MS	3	0.37	1.50E-07	1.72	--	--	--
Fatty Acyls														
Leukotriene C5	$C_{30}H_{45}N_3O_9S$	645.2725	13.71	6	$[M+Na]^+$	4.94	GM-LC-QTOF-MS	4	0.58		1.69	--	--	--
MAG 18:2/Dimethyl-pentyl-furandecanoic acid	$C_{21}H_{38}O_4$/$C_{21}H_{36}O_3$	336.2671	11.55	3/4	$[M+H-H_2O]^+$/$[M+H]^+$	8.35	GM-LC-QTOF-MS	4	0.65	--	1.27	--	--	--
Oxooctanoyl-CoA/Trimethylhepta-dienoyl-CoA	$C_{29}H_{48}N_7O_{18}P_3S$/$C_{31}H_{48}N_7O_{19}P_3S$	929.1832	0.75	3/1	$[M+Na]^+$/$[M+H-H_2O]^+$	4.81	GM-LC-QTOF-MS	3	1.07	5.24E-02ǂ	1.24	1.23	6.09E-03ǂ	2.64
Tetracosahexaenoic acid	$C_{24}H_{36}O_2$	356.2719	10.79	3	$[M+H]^+$	12.93	GM-LC-QTOF-MS	3	1.18	5.24E-02ǂ	--	--	--	--
Ketoleucine	$C_6H_{10}O_3$	130.1418	9.06	-	-	2.27	GC-QTOF-MS	1	--	--	--	1.44	4.33E-04ǂ	--
Elaidic acid	$C_{18}H_{34}O_2$	282.4614	20.26	-	-	6.98	GC-QTOF-MS	1	--	--	--	1.40	--	1.28
Caproic acid	$C_6H_{12}O_2$	116.1583	6.91	-	-	6.30	GC-QTOF-MS	1	--	--	--	1.33	9.21E-04ǂ	1.73
Lauric acid	$C_{12}H_{24}O_2$	200.3178	14.61	-	-	4.51	GC-QTOF-MS	1	1.11	4.89E-02	--	--	--	--
Linoleic acid	$C_{18}H_{32}O_2$	280.4472	20.22	-	-	8.86	GC-QTOF-MS	1	1.31	3.77E-03ǂ	--	4.62	2.27E-04ǂ	--
Myristic acid	$C_{14}H_{28}O_2$	228.3709	16.74	-	-	4.19	GC-QTOF-MS	1	1.17	2.91E-02	--	--	--	--
Stearic acid	$C_{18}H_{36}O_2$	284.4772	20.46	-	-	6.60	GC-QTOF-MS	1	--	--	--	1.10	--	1.24
Dehydrododecanoyl-CoA	$C_{33}H_{56}N_7O_{17}P_3S$	929.2548	7.29	1	$[M+H-H_2O]^+$	5.09	GL-LC-QTOF-MS	4	0.74	--	1.02	1.68	--	1.50

Hydroxybutyryl-CoA	$C_{25}H_{42}N_7O_{18}P_3S$	870.1732	6.42	6	$[M+NH_4]^+$	6.05	GL-LC-QTOF-MS	2	0.24	7.85E-07[‡]	1.97	--	--	--
Oleoyl-CoA	$C_{39}H_{68}N_7O_{17}P_3S$	1069.322	12.96	6	$[M+K]^+$	3.67	GL-LC-QTOF-MS	2	0.35	1.74E-04[‡]	1.34	--	--	--
Oxo-dodecanoyl-CoA	$C_{33}H_{56}N_7O_{18}P_3S$	985.249	12.43	6	$[M+Na]^+$	4.67	GL-LC-QTOF-MS	3	0.33	3.69E-05	1.46	--	--	--
Stearoyl-CoA	$C_{39}H_{70}N_7O_{17}P_3S$	1071.322	12.96	10	$[M+K]^+$	4.25	GL-LC-QTOF-MS	4	0.36	8.63E-04	1.29	--	--	--
Carnitine														
Tetradecadiencarnitine	$C_{21}H_{37}NO_4$	367.2729	9.60	3	$[M+H]^+$	9.31	GM-LC-QTOF-MS	2	--	--	--	1.21	--	1.27
Hexadecadienylcarnitine	$C_{25}H_{45}NO_4$	423.3356	11.74	3	$[M+H]^+$	8.71	GM-LC-QTOF-MS	2	0.49		1.12	--	--	--
Decenoylcarnitine	$C_{17}H_{31}NO_4$	313.2255	7.72	2	$[M+H]^+$	9.79	GM-LC-QTOF-MS	2	--	--	--	1.32	--	1.17
Glycerolipids														
2-Palmitoylglycerol	$C_{19}H_{38}O_4$	330.5026	23.08	-	-	17.61	GC-QTOF-MS	1	1.06	3.96E-02	--	--	--	--
DG 34:1	$C_{37}H_{70}O_5$	576.5152	14.79	7	$[M+H-H_2O]^+$	1.02	GL-LC-QTOF-MS	4	0.74	--	1.15	--	--	--
DG 34:2	$C_{39}H_{66}O_5$	614.4895	12.45	2	$[M+H]^+/[M+Na]^+$	2.42	GL-LC-QTOF-MS	3	0.79	--	1.10	--	--	--
DG 34:3	$C_{37}H_{66}O_5$	572.4817	14.74	3	$[M+H-H_2O]^+$	1.05	GL-LC-QTOF-MS	4	0.77	--	1.02	--	--	--
DG 36:1	$C_{39}H_{74}O_5$	604.5441	9.78	2	$[M+H-H_2O]^+$	2.33	GL-LC-QTOF-MS	4	--	--	--	0.83	--	1.17
DG 36:3	$C_{39}H7_{0}O_5$	600.5129	15.2	3	$[M+H-H_2O]^+$	1.03	GL-LC-QTOF-MS	4	0.80	--	1.06	0.79	--	1.20
DG 36:4	$C_{39}H_{68}O_5$	616.5081	11.59	3	$[M+H]^+/[M+Na]^+/[M+NH_4]^+$	2.14	GL-LC-QTOF-MS	2	0.65	3.37E-02	1.34	--	--	--
DG 46:0	$C_{49}H_{96}O_5$	802.6817	13.73	1	$[M+K]^+$	1.12	GL-LC-QTOF-MS	3	1.68	1.78E-02	--	--	--	--
TG 47:1	$C_{50}H_{96}O_5$	814.6833	12.91	3	$[M+K]^+$	0.83	GL-LC-QTOF-MS	4	0.89	--	1.21	0.71	2.09E-02	2.40
TG 50:0	$C_{53}H_{104}O_5$	858.749	14.58	6	$[M+K]^+$	1.51	GL-LC-QTOF-MS	4	0.76	--	1.09	--	--	--
TG 51:4	$C_{54}H_{96}O_6$	857.7477	14.58	1	$M+NH_4/[M+Na]^+$	0.58	GL-LC-QTOF-MS	2	0.76	--	1.10	--	--	--
TG 52:3	$C_{55}H_{100}O_6$	873.7815	15.2	4	$[M+H]^+/M+NH_4$	0.19	GL-LC-QTOF-MS	2	0.94	--	1.16	0.90	--	1.11
TG 52:4	$C_{55}H_{98}O_6$	876.721	14.79	4	$[M+H]^+/[M+Na]^+/[M+NH_4$	0.51	GL-LC-QTOF-MS	2	0.85	--	1.01	--	--	--
TG 52:5	$C_{55}H_{96}O_6$	852.7222	14.46	2	$[M+H]^+/[M+Na]^+/[M+NH_4]^+/[M+K]^+$	0.68	GL-LC-QTOF-MS	2	0.78	--	1.14	--	--	--
TG 52:6	$C_{55}H_{94}O_6$	872.6883	14.23	2	$[M+Na]^+/[M+NH4]^+$	1.08	GL-LC-QTOF-MS	2	0.78	--	1.02	--	--	--
TG 53:3	$C_{56}H_{102}O_6$	892.7512	15.47	2	$[M+Na]^+$	0.68	GL-LC-QTOF-MS	2	--	--	--	0.85	--	1.14
TG 53:4	$C_{56}H_{100}O_6$	885.78	14.97	2	$[M+NH_4]^+/[M+Na]^+$	1.16	GL-LC-QTOF-MS	2	0.78	--	1.08	--	--	--
TG 53:5	$C_{56}H_{98}O_6$	883.7626	14.38	2	$[M+Na]^+$	1.42	GL-LC-QTOF-MS	2	0.83	--	1.01	--	--	--
TG 54:0	$C_{57}H_{112}O_5$	914.808	15.2	2	$[M+K]^+$	1.69	GL-LC-QTOF-MS	3	--	--	--	0.89	--	1.13
TG 54:1	$C_{57}H_{110}O_5$	912.7924	14.79	2	$[M+K]^+$	1.60	GL-LC-QTOF-MS	2	0.84	--	1.23	--	--	--
TG 54:2	$C_{57}H_{10}8O_5$	910.7758	14.46	1	$[M+K]^+$	1.01	GL-LC-QTOF-MS	2	0.79	--	1.24	--	--	--
TG 54:4	$C_{57}H_{102}O_6$	899.797	15.2	4	$[M+NH_4]^+$	0.36	GL-LC-QTOF-MS	2	0.79	--	1.14	0.79	--	1.27
TG 54:5	$C_{57}H_{100}O_6$	902.7353	14.79	2	$[M+H]^+/[M+Na]^+/[M+NH_4]^+$	0.62	GL-LC-QTOF-MS	2	0.81	--	1.16	--	--	--
TG 54:6	$C_{57}H_{98}O_6$	895.7649	14.63	3	$[M+Na]^+/[M+NH_4]^+/[M+K]^+$	0.51	GL-LC-QTOF-MS	2	0.78	--	1.12	0.78	--	1.03

TG 55:4	$C_{58}H_{104}O_6$	913.8093	15.2	0	$[M+NH_4]^+$	1.68	GL-LC-QTOF-MS	4	--	--	--	0.89	--	1.13
TG 55:8	$C_{58}H_{96}O_6$	888.7199	14.59	0	$[M+H]^+$	1.00	GL-LC-QTOF-MS	4	0.84	--	1.01	--	--	--
TG 56:2	$C_{59}H_{110}O_6$	952.7842	13.49	2	$[M+K]^+$	5.86	GL-LC-QTOF-MS	4	0.63	--	1.23	--	--	--
TG 56:3	$C_{59}H_{110}O_5$	936.7916	14.63	1	$[M+K]^+$	1.31	GL-LC-QTOF-MS	3	--	--	--	0.84	--	1.03
TG 56:5	$C_{59}H_{104}O_6$	946.7415	15.53	3	$[M+K]^+/[M+Na]^+$	1.01	GL-LC-QTOF-MS	2	--	--	--	0.77	--	1.43
TG 56:6	$C_{59}H_{102}O_6$	923.7958	14.99	2	$[M+Na]^+/[M+NH_4]^+$	0.29	GL-LC-QTOF-MS	2	--	--	--	0.71	--	1.52
TG 56:7	$C_{59}H_{100}O_6$	926.7356	14.61	0/3	$[M+Na]^+/[M+NH_4]^+$	0.77	GL-LC-QTOF-MS	2	0.81	--	1.04	0.78	--	1.32
TG 58:3	$C_{61}H_{114}O_5$	964.8228	14.99	1	$[M+K]^+$	1.16	GL-LC-QTOF-MS	3	0.86	--	1.03	0.78	--	1.55
TG 58:4	$C_{61}H_{112}O_5$	962.8073	14.61	1	$[M+K]^+$	1.24	GL-LC-QTOF-MS	2	0.83	--	1.04	0.80	--	1.29
TG 58:7	$C_{61}H_{104}O_6$	949.8113	14.97	2	$[M+NH_4]^+/[M+Na]^+$	0.53	GL-LC-QTOF-MS	2	--	--	--	0.76	--	1.26
TG 60:11	$C_{63}H_{102}O_5$	976.734	15.2	6	$[M+K]^+$	0.81	GL-LC-QTOF-MS	2	--	--	--	0.89	--	1.14
Glycerophospholipids														
CDP-DG 40:4	$C_{52}H_{89}N_3O_{15}P_2$	1079.5543	11.86	4	$[M+Na]^+$	19.15	GM-LC-QTOF-MS	4	0.81		2.38	--	--	--
Glycerophospho Palmitoyl Ethanolamine	$C_{21}H_{44}NO_7P$	453.2867	12.08	4	$[M+H]^+$	9.59	GM-LC-QTOF-MS	2	0.82	--	1.34	0.79	8.22E-02[ł]	--
LPC 15:0	$C_{23}H_{48}NO_7P$	481.3180	13.63	3	$[M+H]^+$	9.72	GM-LC-QTOF-MS	2	0.87		1.77	0.74	9.29E-03[ł]	1.90
LPC 16:0	$C_{24}H_{50}NO_7P$	477.3227	12.15	2	$[M+H-H_2O]^+$	8.30	GM-LC-QTOF-MS	2	0.53		2.03	--	--	--
LPC 17:1/LPE 20:1	$C_{25}H_{50}NO_7P$	507.3336	11.81	3	$[M+H]^+$	9.01	GM-LC-QTOF-MS	3	0.86	--	1.38	--	--	--
LPC 18:0	$C_{26}H_{54}NO_7P$	545.3496	12.16	8	$[M+Na]^+$	6.72	GM-LC-QTOF-MS	2	0.97	--	1.29	--	--	--
LPC 18:1	$C_{26}H_{52}NO_7P$	521.3493	12.71	3	$[M+H]^+$	10.50	GM-LC-QTOF-MS	2	0.98	--	1.42	--	--	--
LPC 18:3	$C_{26}H_{48}NO_7P$	517.3177	11.00	3	$[M+H]^+$	8.19	GM-LC-QTOF-MS	2	--	--	--	1.57		1.84
LPC 20:2	$C_{28}H_{54}NO_7P$	547.3653	12.95	4	$[M+H]^+$	9.07	GM-LC-QTOF-MS	2	0.79		1.99	--	--	--
LPC 20:3/LPC 18:0	$C_{28}H_{52}NO_7P/C_{26}H_{54}NO_7P$	545.3497	11.61	5	$[M+H]^+/[M+Na]^+$	9.09	GM-LC-QTOF-MS	3	0.87		1.21	--	--	--
LPC 22:4/PC O-20:1	$C_{30}H_{54}NO_7P/C_{28}H_{56}NO_7P$	571.3647	12.73	4	$[M+H]^+/[M+Na]^+$	8.27	GM-LC-QTOF-MS	3	0.27		1.70	--	--	--
LPC 22:5	$C_{30}H_{52}NO_7P$	569.3492	11.96	3	$[M+H]^+$	7.50	GM-LC-QTOF-MS	2	0.79	--	1.28	--	--	--
LPC O-16:0	$C_{24}H_{52}NO_6P$	481.3544	12.52	4	$[M+H]^+$	10.49	GM-LC-QTOF-MS	2	0.96	--	1.73	--	--	--
PA O-31:2	$C_{34}H_{65}O_7P$	598.4331	12.15	4	$[M+H-H_2O]^+$	12.90	GM-LC-QTOF-MS	4	0.85	6.23E-02ł	2.12	--	--	--
PC O-20:1	$C_{28}H_{56}NO_7P$	549.3804	14.00	3	$[M+H]^+$	8.50	GM-LC-QTOF-MS	3	0.77	--	1.30	0.86		1.32
Thio-PAF	$C_{26}H_{54}NO_6PS$	561.3210	13.71	3	$[M+Na]^+$	7.92	GM-LC-QTOF-MS	4	0.63		1.58	--	--	--
CL 65:4	$C_{74}H1_{36}O_{17}P_2$	1396.88	14.84	0	$[M+K]^+$	7.84	GL-LC-QTOF-MS	4	0.30	9.98E-06	2.04	--	--	--
LPA 20:4	$C_{23}H_{39}O_7P$	458.2417	2.67	2	$[M+H]^+$	1.70	GL-LC-QTOF-MS	3	0.88	--	1.19	--	--	--
LPC 16:0	$C_{24}H_{50}NO_7P$	495.3337	2.67	4	$[M+H]^+$	1.57	GL-LC-QTOF-MS	2	0.85	--	1.28	--	--	--
LPC 18:0	$C_{26}H_{54}NO_7P$	523.3652	3.94	4	$[M+H]^+$	1.70	GL-LC-QTOF-MS	2	0.81	4.40E-02	1.57	--	--	--

LPC 20:4	$C_{28}H_{50}NO_7P$	543.3331	2.11	2	$[M+H]^+$	2.04	GL-LC-QTOF-MS	2	0.83	--	1.26	--	--	--
LPC O-16:1	$C_{24}H_{50}NO_6P$	479.338	3.2	2	$[M+H]^+$	3.92	GL-LC-QTOF-MS	2	0.82	--	1.50	--	--	--
PC 34:0	$C_{42}H_{84}NO_8P$	761.5946	12.38	2	$[M+H]^+$	1.19	GL-LC-QTOF-MS	2	0.86	4.40E-02	1.32	0.78	--	1.87
PC 38:5	$C_{46}H_{82}NO_8P$	807.5798	8.88	3	$[M+H]^+$	0.63	GL-LC-QTOF-MS	2	--	--	--	0.86	--	1.34
PC 40:7	$C_{48}H_{82}NO_8P$	831.5785	8.57	1	$[M+H]^+$	1.08	GL-LC-QTOF-MS	2	0.84	--	1.30	0.85	--	1.17
PC O-18:0	$C_{26}H_{54}NO_7P$	523.365	3.67	3	$[M+H]^+$	1.55	GL-LC-QTOF-MS	2	0.81	4.05E-02	1.62	--	--	--
PC O-34:2	$C_{42}H_{82}NO_7P$	743.5834	11.69	1	$[M+H]^+$	1.37	GL-LC-QTOF-MS	2	--	--	--	0.74	4.44E-02	2.35
PC O-36:1	$C_{44}H_{88}NO_7P$	795.6157	12.29	6	$[M+Na]^+$	1.21	GL-LC-QTOF-MS	2	0.88	--	1.10	--	--	--
PC O-38:4	$C_{46}H_{86}NO_7P$	795.6139	11.78	0	$[M+H]^+$	1.60	GL-LC-QTOF-MS	2	0.87	4.05E-02	1.28	0.84	--	1.60
PC O-40:2	$C_{48}H_{94}NO_7P$	865.6321	8.88	0	$[M+K]^+$	1.50	GL-LC-QTOF-MS	2	0.92	--	1.02	0.87	--	1.31
PE 36:4	$C_{41}H_{74}NO_8P$	739.5166	8.97	3	$[M+H]^+$	1.34	GL-LC-QTOF-MS	2	--	--	--	0.65	2.88E-02	1.80
PE 38:4	$C_{43}H_{78}NO_8P$	767.5477	11.2	2	$[M+H]^+$	1.06	GL-LC-QTOF-MS	2	--	--	--	0.66	2.88E-02	1.79
PE 38:6	$C_{43}H_{74}NO_8P$	763.5164	8.62	2	$[M+H]^+$	1.02	GL-LC-QTOF-MS	2	--	--	--	0.62	--	1.66
PE 40:6	$C_{45}H_{78}NO_8P$	791.5482	10.67	3	$[M+H]^+$	1.30	GL-LC-QTOF-MS	2	--	--	--	0.72	--	1.29
PG 43:2	$C_{49}H_{93}O_{10}P$	894.6349	13.25	3	$[M+Na]^+$	1.87	GL-LC-QTOF-MS	3	--	--	--	0.96	--	1.02
PS 39:0	$C_{45}H_{88}NO_{10}P$	850.6353	12.91	6	$[M+NH_4]^+$	4.56	GL-LC-QTOF-MS	3	--	--	--	0.75	3.71E-02	2.00
Organoheterocyclic compounds														
Biliverdin	$C_{33}H_{34}N_4O_6$	582.2484	9.10	2	$[M+H]^+$	13.90	GM-LC-QTOF-MS	3	--	--	--	1.94		1.17
Formyl Indole	C_9H_7NO	145.0529	2.51	5	$[M+H]^+$	9.58	GM-LC-QTOF-MS	2	0.58	--	1.04	--	--	--
Lipoyllysine	$C_{14}H_{26}N_2O_3S_2$	372.0926	6.42	4	$[M+K]^+$	3.87	GL-LC-QTOF-MS	4	0.36	1.38E-05	1.75	--	--	--
Hemin	$C_{34}H_{32}ClFeN_4O_4$	668.168	5.16	6	$[M+NH_4]^+$	6.18	GL-LC-QTOF-MS	4	0.46	1.70E-04	1.78	--	--	--
Methylxanthine/Methylmalate	$C_6H_6N_4O_2/C_5H_8O_5$	148.0379	4.44	1/8	$[M+H-H_2O]^+/[M+H]^+$	7.70	GL-LC-QTOF-MS	3	0.47	3.80E-05	1.84	--	--	--
Tryptophanol/Dehydroxycarnitine/Aminoheptanoic acid	$C_{10}H_{11}NO/C_7H_{15}NO_2$	183.0659	2.67	3	$[M+Na]^+/[M+K]^+$	2.19	GL-LC-QTOF-MS	3	0.84	--	1.26	--	--	--
Indoles and derivatives														
Indoleacrylic acid	$C_{11}H_9NO_2$	187.0633	3.24	3	$[M+H]^+$	10.81	GM-LC-QTOF-MS	2	--	--	--	1.54	--	1.13
Indolelactic acid	$C_{11}H_{11}NO_3$	187.0634	2.51	2	$[M+H-H_2O]^+$	9.51	GM-LC-QTOF-MS	2	0.97	--	1.13	1.27	--	1.86
Sphingolipids														
C17 Sphinganine	$C_{17}H_{37}NO_2$	287.2832	9.65	5	$[M+H]^+$	9.78	GM-LC-QTOF-MS	2	--	--	--	0.69	--	1.18
Ganglioside GD3 (41:1)	$C_{75}H_{135}N_3O_{29}$	1541.9198	12.15	1	$[M+H]^+$	7.91	GM-LC-QTOF-MS	4	0.81	1.46E-02	2.25	--	--	--
Hex(4)-HexNAc(2)-NeuAc(2)-Cer 36:1;O2	$C_{98}H_{171}N_5O_{49}$	2184.0974	9.09	2	$[M+H-H_2O]^+$	8.94	GM-LC-QTOF-MS	4	0.40	--	1.12	--	--	--
Hex(4)-HexNAc(3)-NeuCer 38:1;O2	$C_{97}H_{171}N_5O_{47}$	2140.0947	9.34	4	$[M+H-H_2O]^+$	15.24	GM-LC-QTOF-MS	4	--	--	--	1.30		1.54
Hex(5)-HexNAc-NeuAc(2)-Cer 38:1;O2	$C_{98}H_{172}N_4O_{49}$	2171.1043	9.30	3	$[M+H-H_2O]^+$	9.59	GM-LC-QTOF-MS	4	--	--	--	1.27		--

NeuAcalpha2-3Galbeta-Cer(34:1)	$C_{51}H_{94}N_2O_{16}$	990.6675	12.15	8	$[M+H]^+$	15.79	GM-LC-QTOF-MS	4	0.92	2.43E-02	2.40	--	--	--
NeuAcHexCer 36:1;O2	$C_{53}H_{98}N_2O_{16}$	1040.6825	12.16	9	$[M+Na]^+$	13.66	GM-LC-QTOF-MS	4	0.93	--	1.85	--	--	--
Phytosphingosine	$C_{18}H_{39}NO_3$	317.2935	9.05	3	$[M+H]^+$	10.20	GM-LC-QTOF-MS	2	0.44	7.33E-04ǂ	--	5.72		1.20
SM 36:2;O2/CerPE 39:2;O2	$C_{41}H_{81}N_2O_6P$	750.5654	16.42	1	$[M+Na]^+$	6.51	GM-LC-QTOF-MS	3	1.06	--	1.06	--	--	--
CerPE 37:1;O2	$C_{39}H_{79}N_2O_6P$	740.5249	8.14	3	$[M+K]^+$	1.33	GL-LC-QTOF-MS	3	0.85	4.32E-02	1.20	0.73	2.83E-02	2.38
Hex2Cer 34:1;O2	$C_{46}H_{87}NO_{13}$	861.619	7.86	2	$[M+H]^+$	1.20	GL-LC-QTOF-MS	2	0.83	3.41E-02	1.11	--	--	--
HexCer 36:1;O3	$C_{42}H_{81}NO_9$	760.6222	8.14	7	$[M+NH_4]^+$	3.00	GL-LC-QTOF-MS	3	0.75	3.41E-02	1.44	0.64	--	2.06
HexCer 42:2;O2	$C_{48}H_{91}NO_8$	809.675	12.93	1	$[M+H]^+$	1.20	GL-LC-QTOF-MS	3	--	--	--	0.95	--	1.12
SM 34:0;O2	$C_{39}H_{81}N_2O_6P$	704.5844	8.81	2	$[M+H]^+$	1.14	GL-LC-QTOF-MS	2	0.87	--	1.08	0.60	2.09E-02	2.14
SM 34:1;O2	$C_{39}H7_{9}N_2O_6P$	702.5696	8.14	4	$[M+H]^+$	1.12	GL-LC-QTOF-MS	2	0.84	3.48E-02	1.37	0.75	--	2.22
SM 36:1;O2	$C_{41}H_{83}N_2O_6P$	730.6004	10.05	3	$[M+H]^+$	1.31	GL-LC-QTOF-MS	2	0.85	6.91E-03	1.32	0.74	--	1.88
SM 40:0;O2/DG 45:0	$C_{45}H_{93}N_2O_6P/C_{48}H_{94}O_5$	788.67	12.92	8/6	$[M+H]^+/[M+K]^+$	1.29	GL-LC-QTOF-MS	4	--	--	--	0.92	--	1.16
SM 40:1;O2	$C_{45}H_{91}N_2O_6P$	786.664	12.92	4	$[M+H]^+$	0.67	GL-LC-QTOF-MS	2	--	--	--	0.91	--	1.18
SM 41:2;O2	$C_{46}H_{91}N_2O_6P$	798.6624	12.72	2	$[M+H]^+$	1.28	GL-LC-QTOF-MS	2	0.86	--	1.17	0.71	1.94E-02	2.48
SM 42:1;O2	$C_{47}H_{95}N_2O_6P$	814.695	13.25	3	$[M+H]^+$	1.03	GL-LC-QTOF-MS	2	--	--	--	0.93	--	1.07
SM 42:2;O2	$C_{47}H_{93}N_2O_6P$	812.6794	12.91	3	$[M+H]^+$	0.68	GL-LC-QTOF-MS	2	0.89	--	1.23	0.73	2.88E-02	2.35
SM 42:3;O2	$C_{47}H_{91}N_2O_6P$	810.6623	12.55	2	$[M+H]^+$	1.03	GL-LC-QTOF-MS	2	0.87	--	1.22	0.71	1.94E-02	2.38
SM 43:2;O2	$C_{48}H_{95}N_2O_6P$	826.6939	13.02	2	$[M+H]^+$	1.27	GL-LC-QTOF-MS	2	0.83	--	1.00	0.67	1.94E-02	2.09
Sterol Lipids														
Chenodeoxyglycocholic acid	$C_{26}H_{43}NO_5$	449.3146	8.16	2	$[M+H]^+$	11.49	GM-LC-QTOF-MS	3	--	--	--	2.64	--	1.01
Glycocholic acid	$C_{26}H_{43}NO_6$	447.2988	8.81	2	$[M+H-H_2O]^+$	11.92	GM-LC-QTOF-MS	4	2.29	8.94E-03	--	8.35	6.09E-03ǂ	--
Hydrocortisone succinate/Dehydrotestosterone glucosiduronic acid	$C_{25}H_{34}O_8$	462.2259	9.65	2	$[M+H]^+$	7.63	GM-LC-QTOF-MS	3	0.16	4.65E-02	--	1.39	--	--
Bilirubins														
Mesobilirubinogen	$C_{33}H_{44}N_4O_6$	592.3260	9.58	1	$[M+H]^+$	14.05	GM-LC-QTOF-MS	3	0.09	1.10E-05	--	0.37	9.91E-03ǂ	--
Urobilinogen	$C_{33}H_{48}N_4O_6$	596.3573	9.59	1	$[M+H]^+$	12.96	GM-LC-QTOF-MS	3	1.49	5.31E-03	--	0.18	6.09E-03ǂ	2.33
Others														
Inosine cyclic phosphate	$C_{10}H_{11}N_4O_7P$	330.0377	0.68	5	$[M+H]^+$	1.73	GL-LC-MS	3	--	--	--	1.11	4.81E-02	2.10
UDP-acetyl-(carboxyvinyl)-glucosamine	$C_{20}H_{29}N_3O_{19}P_2$	659.0740	0.73	3	$[M+H-H_2O]^+$	1.39	GM-LC-QTOF-MS	4	--	--	--	1.12	6.09E-03ǂ	2.88

RT: retention time; [a]CV, coefficient of variation in the metabolites in the QC samples; [b]Identification level: Level 1 Structure confirmed, Level 2 Structure probable, Level 3 Unequivocal molecular formula (s), Level 4 Exact mass; [c]Fold Change, change in the abundance of the specified comparison calculated as (case/control); [d]p valueǂ corresponding to the *p* values calculated by the Benjamini-Hochberg false discovery rate post hoc correction (FDR < 0.05); [e]VIP, variable importance in projection. GM: global metabolomics, GL: global lipidomics, LC: liquid chromatography, GC: gas chromatography, QTOF-MS: quadrupole time-of-flight mass spectrometer.

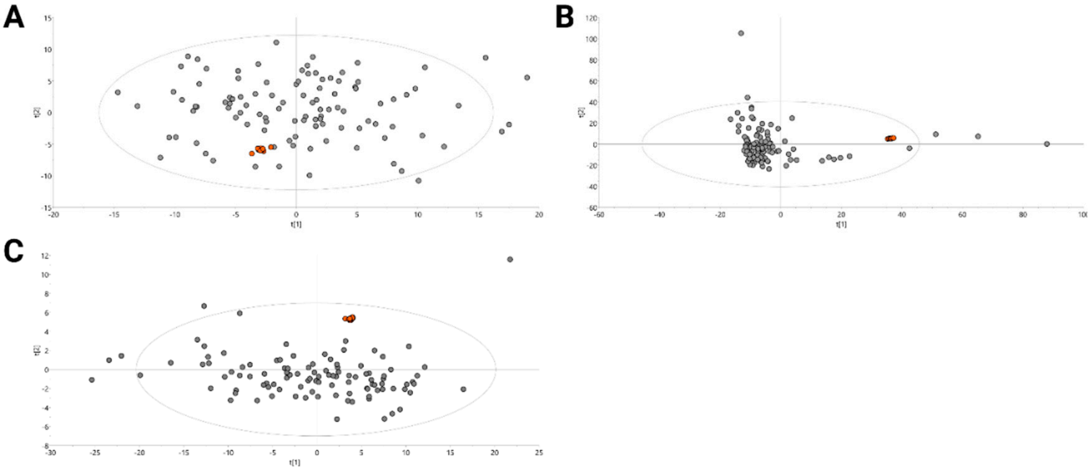

Figure S1. PCA models for metabolic and lipidomic analysis. (**A**) GM(+): R^2: 0.673, (**B**) GL(+): R^2: 0.928; (**C**) GC: R^2: 0.71. Dots in orange denote quality control, gray dots correspond to samples.

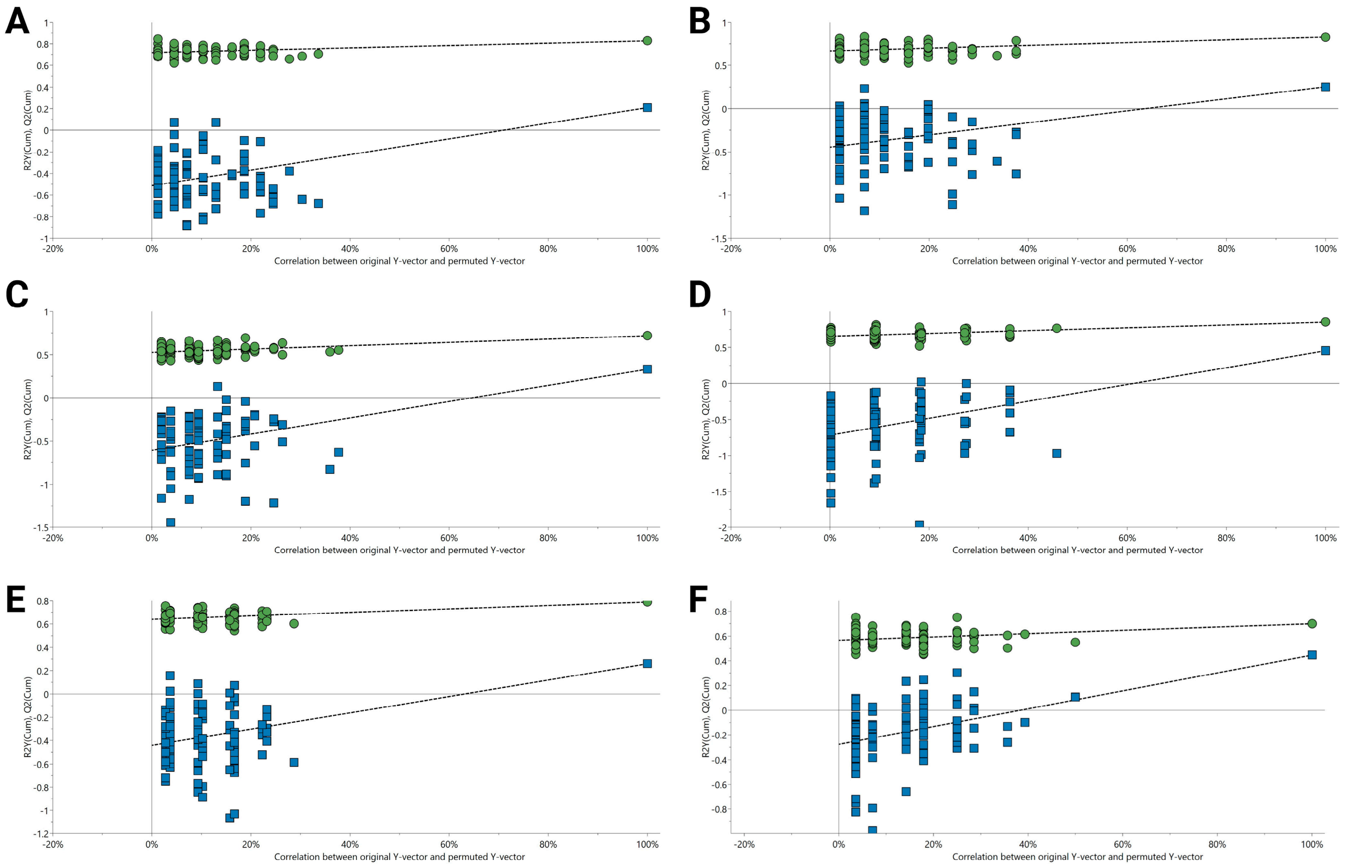

Figure S2. OPLS-DA permutation test plots. (**A,C,E**): BPHvsPCa, (**B,D,F**): PCavsMET. (**A,B**) GM(+), (**C,D**) GL(+), (**E,F**) GC. Dots in green correspond to R^2, squares in blue denote Q^2.

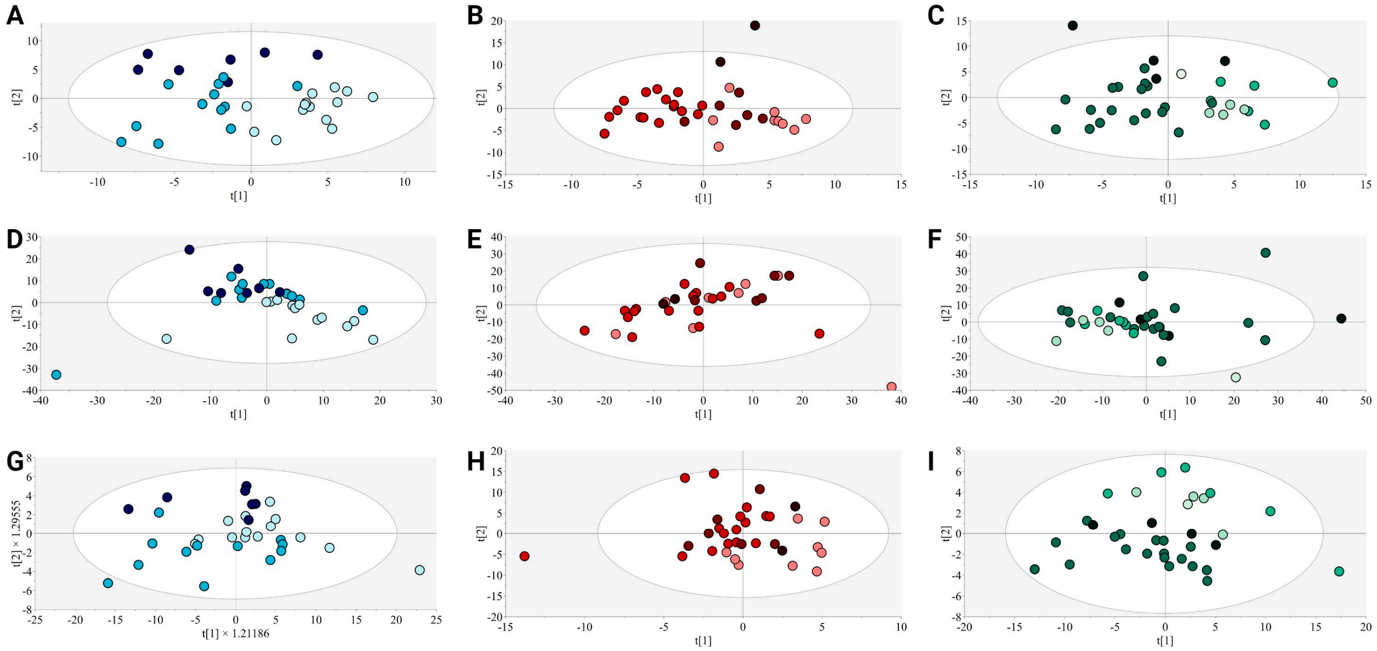

Figure S3. PLSDA Models for comparisons regarding PSA, TNM, and Gleason score classifications.

(**A–C**): GM(+). (**D–F**): GL(+). (**G–I**): GC. (**A,D,G**): PSA classifications. (**B,E,H**): Gleason score, (**C,F,I**): TNM score. (**A**) R^2: 0.551, Q^2: -0.21, cv-ANOVA: 1. (**B**) R^2: 0.479, Q^2: -0.125, cv-ANOVA: 0.96. (**C**) R^2: 0.322, Q^2: -0.05, cv-ANOVA: 1. (**D**) R^2: 0.255, Q^2: -0.045, cv-ANOVA: 0.89. (**E**) R^2: 0.491, Q^2: -0.159, cv-ANOVA: 1. (**F**) R^2: 0.124, Q^2: -0.118, cv-ANOVA: 1. (**G**) R^2: 0.366, Q^2: -0.088, cv-ANOVA: 1. (**H**) R^2: 0.191, Q^2: -0.099, cv-ANOVA: 0.85. (**I**) R^2: 0.248, Q^2: 0.0671, cv-ANOVA: 1. The points classify the samples into descending color gradients as follows: Blue gradient (PSA): >20, 10-20, <10; Red gradient (Gleason Score): 9, 8, 7, 6; Green gradient (TNM Score): T2c, T2b, T2a, T1c, T1b.
